# Supplementary material for: Comparative analysis of two paradigm bacteriophytochromes reveals opposite functionalities in two-component signaling
Source: Nat Commun. 2021 Jul 20;12:4394. doi: 10.1038/s41467-021-24676-7 (PMC8292422; doi:10.1038/s41467-021-24676-7)
Supplement: Supplementary file 1 — Supplementary Information [file 41467_2021_24676_MOESM1_ESM.pdf]

*Comparative analysis of two paradigm bacteriophytochromes reveals opposite functionalities in two-component signaling*

**Elina Multamäki[1], Rahul Nanekar[2], Dmitry Morozov[3], Topias Lievonen[2], David Golonka[4], Weixiao Yuan Wahlgren[5], Brigitte Stucki-Buchli[2], Jari Rossi[1], Vesa P. Hytönen[6], Sebastian Westenhoff[5], Janne A. Ihalainen[2,*], Andreas Möglich[4], Heikki Takala[1,2,*]**

[1] Faculty of Medicine, Anatomy, University of Helsinki, 00014 Helsinki, Finland
[2] Nanoscience Center, Department of Biological and Environmental Science, University of Jyvaskyla, 40014 Jyvaskyla, Finland
[3] Nanoscience Center, Department of Chemistry, University of Jyvaskyla, 40014 Jyvaskyla, Finland
[4] Lehrstuhl für Biochemie, Universität Bayreuth, 95447 Bayreuth, Germany
[5] University of Gothenburg, Department of Chemistry and Molecular Biology, 40530 Gothenburg, Sweden
[6] Faculty of Medicine and Health Technology, BioMediTech, Tampere University, 33520 Tampere, Finland; Fimlab Laboratories, 33520 Tampere, Finland

*To whom correspondence should be addressed: Heikki Takala: Department of Biological and Environmental Science, University of Jyvaskyla, FI-40014, Jyvaskyla; E-mail: heikki.p.takala@jyu.fi; Janne Ihalainen: Department of Biological and Environmental Science, University of Jyvaskyla, FI-40014, Jyvaskyla; E-mail: janne.ihalainen@jyu.fi

## SUPPLEMENTARY DATA

Supplementary Data include Supplementary Figures 1–9, Supplementary Table 1 with their legends, and Supplementary References.

## TABLE OF CONTENTS

Supplementary Figure 1 .......... 2
Supplementary Figure 2 .......... 4
Supplementary Figure 3 .......... 6
Supplementary Figure 4 .......... 7
Supplementary Figure 5 .......... 9
Supplementary Figure 6 .......... 11
Supplementary Figure 7 .......... 12
Supplementary Figure 8 .......... 13
Supplementary Figure 9 .......... 15
Supplementary Table 1 .......... 16
Supplementary References .......... 17

**Supplementary Figure 1**

**a**

| Sample | $A_{750/700}$ | $\tau_1$ (min) | $A_1$ (%) | $\tau_2$ (min) | $A_2$ (%) | $\tau_3$ (min) | $A_3$ (%) |
|---|---|---|---|---|---|---|---|
| DrBphP | 1.25 | 0.8 | 18 | 18 | 24 | 480 | 59 |
| DrBphP+DrRR | 1.16 | 0.7 | 19 | 19 | 34 | 140 | 48 |
| Agp1 | 1.31 | 2.3 | 18 | 13 | 49 | 240 | 33 |
| Agp1+AtRR1 | 1.40 | 2.2 | 12 | 13 | 48 | 250 | 40 |
| Chimera | 1.34 | 1.2 | 6 | 26 | 42 | 390 | 52 |
| Chimera+AtRR1 | 1.31 | 2.0 | 7 | 27 | 39 | 330 | 54 |

**b**

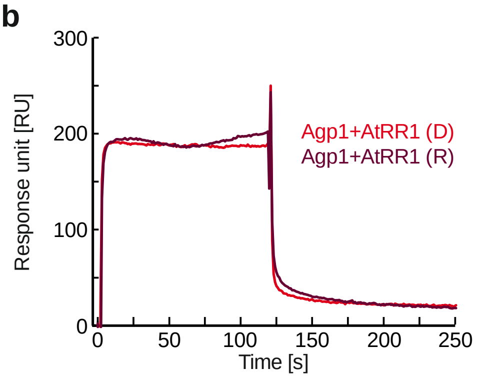


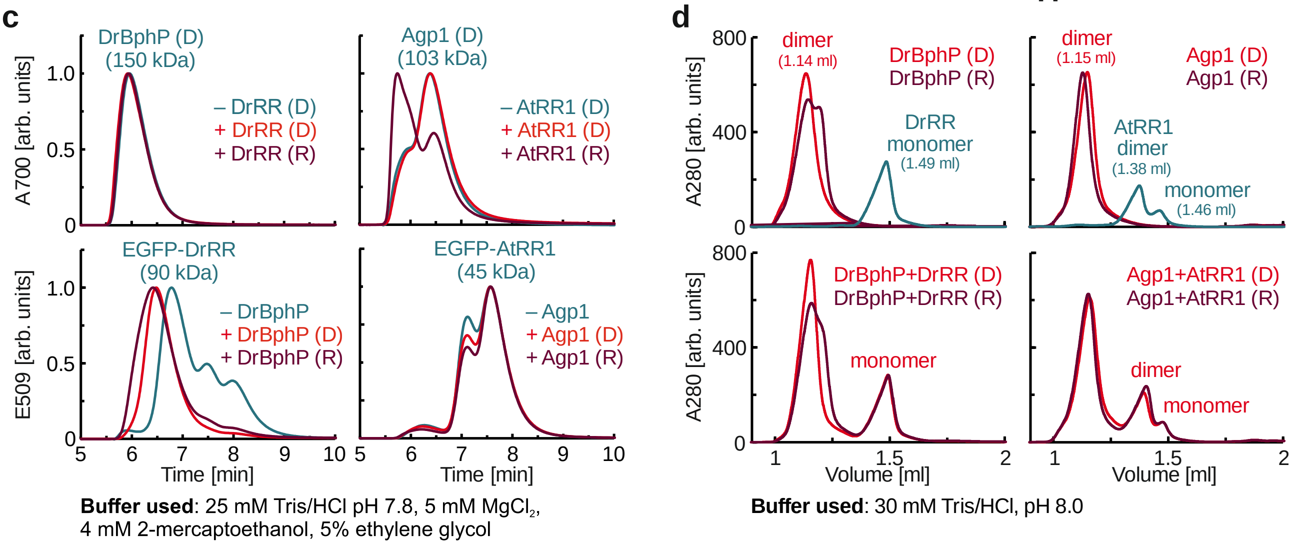


**Supplementary Figure 1**. Evaluation of dark reversion data, Agp1measurements with surface plasmon resonance (SPR), and additional size-exclusion chromatography of BphP/RR pairs. Source data are provided as a Source Data file.

**a**. The dark reversion data (Figure 1b) were approximated with an exponential fits. The table summarizes the time constants ($\tau_n$) and decay amplitudes ($A_n$) of the reversion with and without response regulator. Satisfactory fits were obtained by using a sum of three decay components, resulting in root mean square error (RMSE) values of 0.003–0.006. $A_{750/700}$ denotes the maximum absorption ratio at $t$ = 0 min. The dark reversion of Agp1 was similar to previously published results, where three components were used [1]. DrBphP however had a faster dark reversion rate and different time constants than reported before [2,3]. We therefore note that the differences in experimental parameters, including the buffer system, may cause the discrepancies in the reversion rates. Although the comparison between other studies is therefore problematic, the reversion rates within this study are fully comparable. Addition of the DrRR affected only the third time constant of the DrBphP reversion, from 480 minutes to 140 minutes and their relative amplitudes, which means that the structural properties of the DrBphP that are responsible for the third reversion component is affected by the DrRR binding.

**b.** Surface plasmon resonance (SPR) measurements of Agp1/AtRR1 interaction pair in dark (D) or after red light illumination (R). The measurement was conducted as in Figure 2c by applying Agp1 (100 µM) to AtRR1-coupled sensor surface.

**c–d**. Size exclusion chromatography (SEC) of DrBphP and Agp1 and their response regulators, with or without EGFP fusion. The values represented in parentheses are molecular weights calculated from retention volumes.

Abbreviations: D = dark sample; R = red-illuminated (655 nm) sample. **c.** The size-exclusion measurements with multiwavelength detection were conducted as previously described [2]. For sample separation, a Nanofilm SEC-250 (300 mm × 4.5 mm) column (Sepax Technologies, Delaware, US) was used with (25 mM Tris/HCl pH 7.8, 5 mM $MgCl_2$, 4 mM 2-mercaptoethanol, 5% ethylene glycol) as a mobile phase. Experiments were executed with 350 µl/min flow-rate using the HPLC VP10 pumping system (Shimadzu Corporation, Kyoto, Japan) at room temperature. The eluant was detected at 1.5625 Hz with a diode array UV-Vis detector (SPD-M10A, Shimadzu) and at 2.00 Hz with a fluorescence detector (RF-10$A_{XL}$, Shimadzu). For each run, 20 µl of sample mixture was injected briefly after pre-illumination with 785 nm (D) or 655 nm (R) light. The protein concentrations used were 6 µM (EGFP-DrRR), 30–50 µM (DrBphP), 1 µM (EGFP-AtRR1), and 60 µM (Agp1). Interacting proteins from *D. radiodurans* (DrBphP and EGFP-DrRR) are on the left, and the proteins from *Agrobacterium fabrum* (Agp1, EGFP-AtRR1) are on right. To exclude spectral overlap between the samples, 700 nm absorption was used for BphP detection (upper panels) and fluorescence with 488/509 nm excitation/emission wavelengths, respectively, were used for EGFP-RR detection (lower panels). The Gel Filtration standard (Bio-Rad) was used according to the manufacturer's instructions. The molecular weight estimates were determined by calculating a standard curve of marker proteins Vitamin B12 (1.35 kDa) myoglobin (17 kDa), ovalbumin (44 kDa), γ-globulin (158 kDa), and thyroglobulin (670 kDa). **d**. Size-exclusion chromatography in (30 mM Tris/HCl, pH 8.0). The experiments were conducted at 4°C with Superdex 200 Increase 3.2/300 (GE Healthcare). Each protein was injected at 10 mg/ml concentration in 30 µl volume. Protein retentions were detected at 280 nm. The void volume (1.00 ml) was verified with Dextran. The weight estimation was not possible due to standard protein instability.

The retention graphs show that in some conditions free DrRR (19.1 kDa) was monomeric (d, and Fig. 2a), whereas free AtRR1 (19.1 kDa) appeared as a mixture of mainly dimers (d, and Fig. 2a). In contrast to the Figure 2a in the main text, free EGFP-DrRR (45.4 kDa) appeared dimeric and EGFP-AtRR1 (45.4 kDa) mainly monomeric in panel c, which may be due to the harsh conditions used in the HPLC experiment.

DrBphP (84.0 kDa) and Agp1 (83.8 kDa) are known to be dimers. Whereas both phytochromes appear dimeric in panel c and d, the molecular weight appears higher in Fig. 2a. Agp1 seems to oligomerize in the HPLC measurement after red (R) illumination (panel c), but presumably remains dimeric in other conditions. Free DrBphP seemed to remain dimeric in panel c and d regardless of the illumination. Panel d shows that DrBphP behaved like previously described in (30 mM Tris, pH 8.0) [2], where red light illumination led to a shoulder that elutes right after the main Pr-state peak.

The retention of EGFP-DrRR was clearly shifted by the addition of DrBphP (c, and Fig. 2a). This was apparent, when EGFP-specific wavelengths were used for detection. With DrRR only, the interaction was not directly visible (d). However, the DrBphP elution peaks were slightly affected by the presence of DrRR, which may indicate DrBphP/DrRR interaction. Agp1 did not affect EGFP-AtRR1 retention (c, and Fig. 2a), or AtRR1 did not affect the retention of the Agp1 (d). The Agp1/AtRR1 interaction was therefore not detected with the SEC.

## Supplementary Figure 2

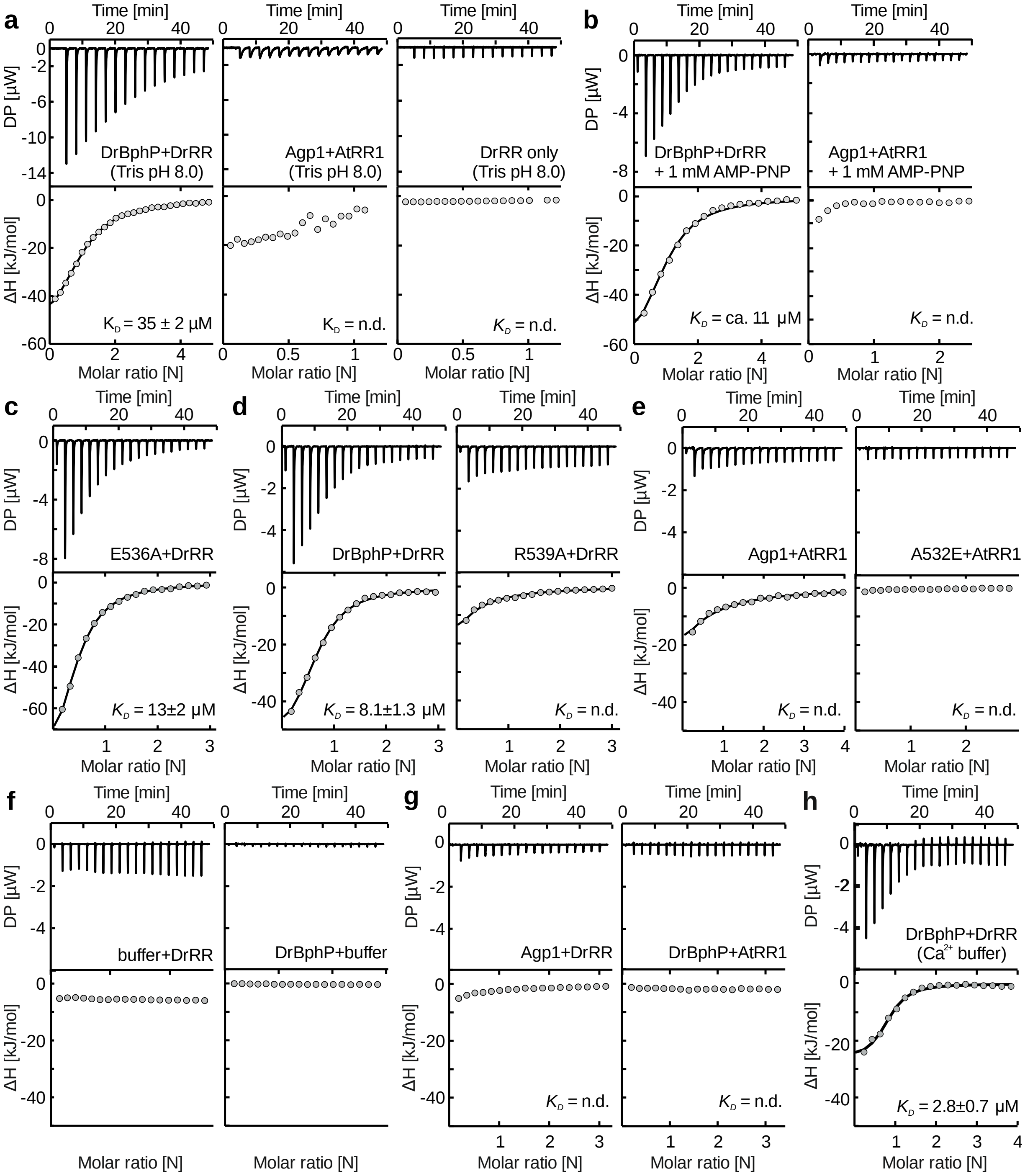


**i**

| | DrBphP (D) + DrRR | DrBphP (R) + DrRR | Agp1 (D) + AtRR1 |
|---|---|---|---|
| **SPR** (*steady-state*)[1] | | | |
| $K_D$ [μM] | 43 ± 8 | 60 ± 7 | *n.d.* |
| $R_{max}$ | 470 ± 30 | 400 ± 20 | *n.d.* |
| Adj. $R^2$ | 0.97 | 0.99 | *n.d.* |
| **SPR** (*kinetic*)[1] | | | |
| $K_D$ [μM] | 6.2 | 15.5 | *n.d.* |
| $R_{max}$ | 195 | 188 | *n.d.* |
| $k_a$ [$M^{-1}s^{-1}$] | 740 | 420 | *n.d.* |
| $k_d$ [$s^{-1}$] | 0.0046 | 0.0065 | *n.d.* |
| $\chi^{2}$ † | 14.9 | 1.16 | *n.d.* |
| **ITC** | | | |
| $K_D$ [μM][2] | 8.1 ± 1.3 | - | *n.d.* |
| $K_D$ [μM][3] | 35 ± 2.0 | - | *n.d.* |

[1] 20 mM HEPES, 300 mM $NaCl_2$, 5 mM $MgCl_2$, 0.10% (v/v) Tween20, pH 7.5

[2] 25 mM Tris/HCl, 5 mM $MgCl_2$, 4 mM 2-mercaptoethanol, 5% ethylene glycol, pH 7.8

[3] 30 mM Tris/HCl, pH 8.0

**Supplementary Figure 2.** Additional measurements with isothermal calorimetry (ITC). **a**. DrRR binding to DrBphP measured in (30 mM Tris/HCl pH 8.0). DrBphP/DrRR interaction occurs with $K_D$ of 35 ± 2 µM, and no Agp1/AtRR1 interaction was detected. The rest of the measurements (panels b–h) were conducted in buffer (50 mM Tris/HCl pH 7.8, 10 mM $MgCl_2$, 8 mM 2-mercaptoethanol, 10% ethylene glycol). **b**. DrBphP/DrRR and Agp1/AtRR1 interactions were barely affected by addition of 1 mM AMP-PNP in the buffer. **c–d**. The interaction between DrBphP mutant E536A was slightly weaker ($K_D$ = 13 ± 2 µM) than the wild-type ($K_D$ = 8.1 ± 1.3 µM). The DrBphP/DrRR interaction is mainly abolished by the R539A mutation. **e**. The binding of Agp1 A532E variant to AtRR1 was too weak to be determined. **f**. The control measurements show that the mixing of protein and buffer results featureless signals. **g**. The phytochromes studied do not cross-interact with each other's response regulators. This indicates that the phosphotransfer reaction between Agp1 and DrRR (Figure 3b) does not require measurable interaction. **h**. The DrBphP/DrRR interaction is slightly stronger if $Mg^{2+}$ is replaced with $Ca^{2+}$. However, this replacement reduces the phosphatase activity (see Supplementary Figure 4f). **i**. Table summarizing the results from SPR and ITC analyses, along with additional fitting parameters. Adjusted $R^2$-values indicate an agreement for the steady-state fits, with the best agreement approaching a value of 1.0. The $\chi^2$ values close to 10 indicate a good kinetic $K_D$ estimation in the case on DrBphP/DrRR interaction. $R_{max}$ values give information on the maximal SPR response in saturating protein concentrations. Source data are provided as a Source Data file.

## Supplementary Figure 3

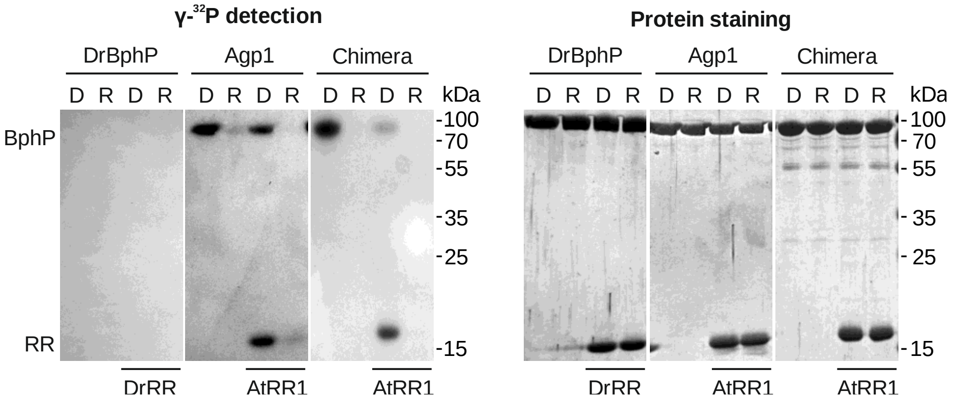


**Supplementary Figure 3.** Extended gels from the radiolabeled ATP assay shown in the Figure 3a in the main text. All shown measurements have been repeated in dependently at least three times. The positions of the molecular weight marker proteins are indicated next to the gels. Source data are provided as a Source Data file, which includes full gels and molecular weight markers.

## Supplementary Figure 4

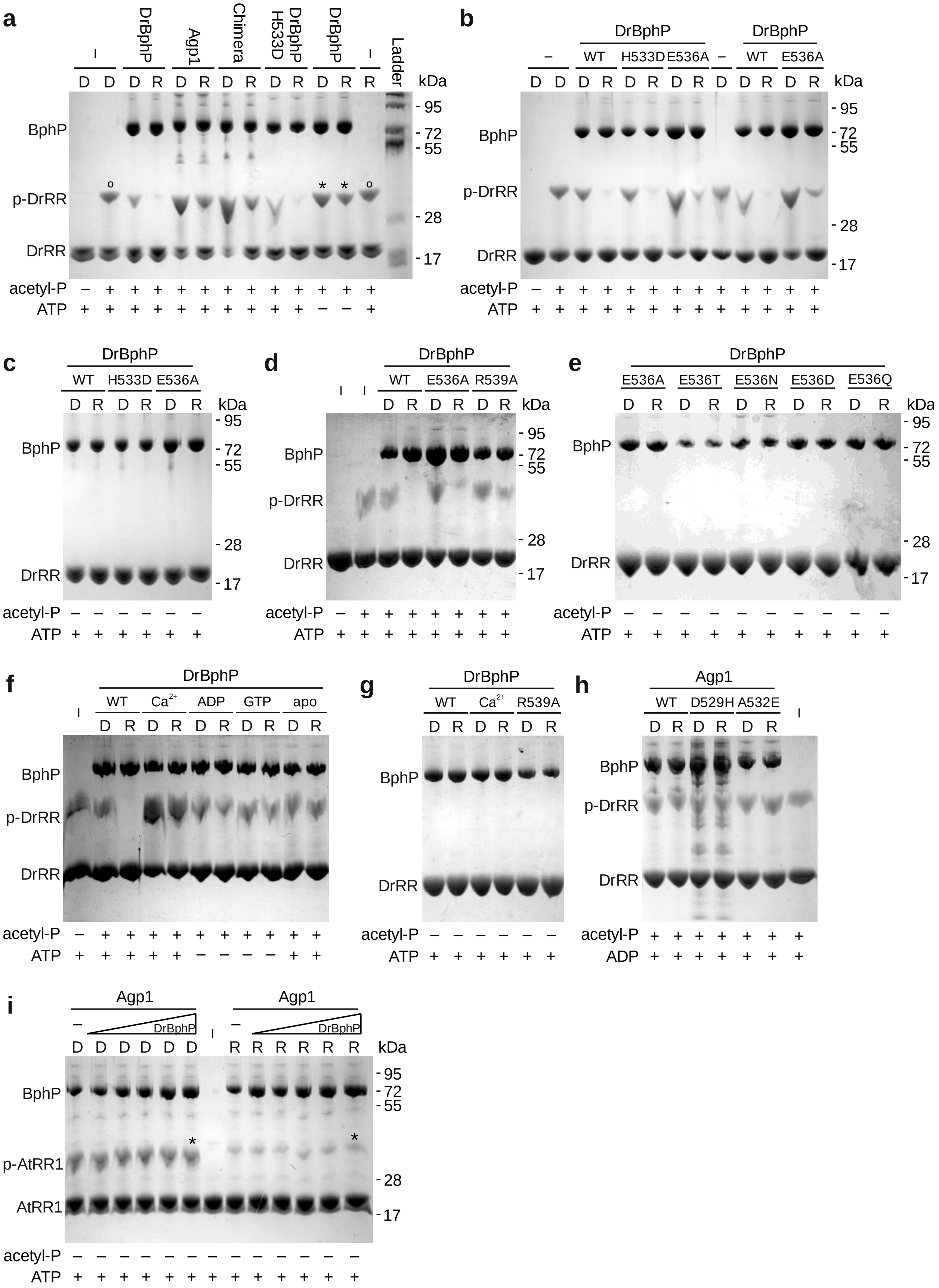

**Supplementary Figure 4.** Supplementary Phos-tag assays. **a**. Extended gel of the kinase assay shown in Figure 3c. The gel also shows that the phosphatase activity of DrBphP is stalled when ATP was excluded from the reaction (*) and that the amount of p-DrRR is not affected by red illumination ($^{O}$). **b**. Extended gel of the results shown in Figure 3d, which also repeats the E536A activity. **c**. Gel showing that wild-type DrBphP and variants H533D and E536A do not have kinase activity. **d**. Gel showing that DrBphP H+4 mutant E536A has lower phosphatase activity than the wild type. In addition the H+7 mutant R539A has even lower phosphatase activity than E536A. **e**. Gel showing that all H+4 variants of DrBphP presented in this paper appear inactive histidine kinases. **f**. The phosphatase activity of DrBphP in different conditions, where $Mg^{2+}$ is replaced with $Ca^{2+}$ (‘$Ca^{2+}$’), or ATP is replaced with ADP (‘ADP’) or GTP (‘GTP’). The phosphatase activity of DrBphP is reduced with ADP, but stalled with $Ca^{2+}$ or GTP. In addition, the DrBphP apoprotein (‘apo’) does not function as light-induced phosphatase. **g**. DrBphP mutant R539A does not function as a kinase, like wild-type DrBphP in $Ca^{2+}$-containing buffer. **h**. Gels showing that Agp1 or its variants D529H and A532E cannot de-phosphorylate p-DrRR, therefore not showing phosphatase activity. **i**. Competition experiment showing no DrBphP cross-activity with AtRR1. In the experiment, increasing amounts of DrBphP were added to the reaction with constant amounts of Agp1 and AtRR1. The highest DrBphP amount used (*) is equal to the Agp1 amount in the reaction. As the level of p-AtRR1 remains constant in all DrBphP concentrations, DrBphP does not seem to de-phosphorylate it. The phytochrome samples are indicated above the gels. The response regulator, its treatment with acetyl phosphate treatment, and the inclusion of ATP are indicated below each gel. The positions of the molecular weight markers are shown in panels a–e, and i, but gels f–h did not include molecular weight markers. Source data (full gels) are provided as a Source Data file. All measurements shown have been repeated independently at least three times, and representative gels are shown here. Abbreviations: D = dark sample; R = red-illuminated (655 nm) sample.

## Supplementary Figure 5

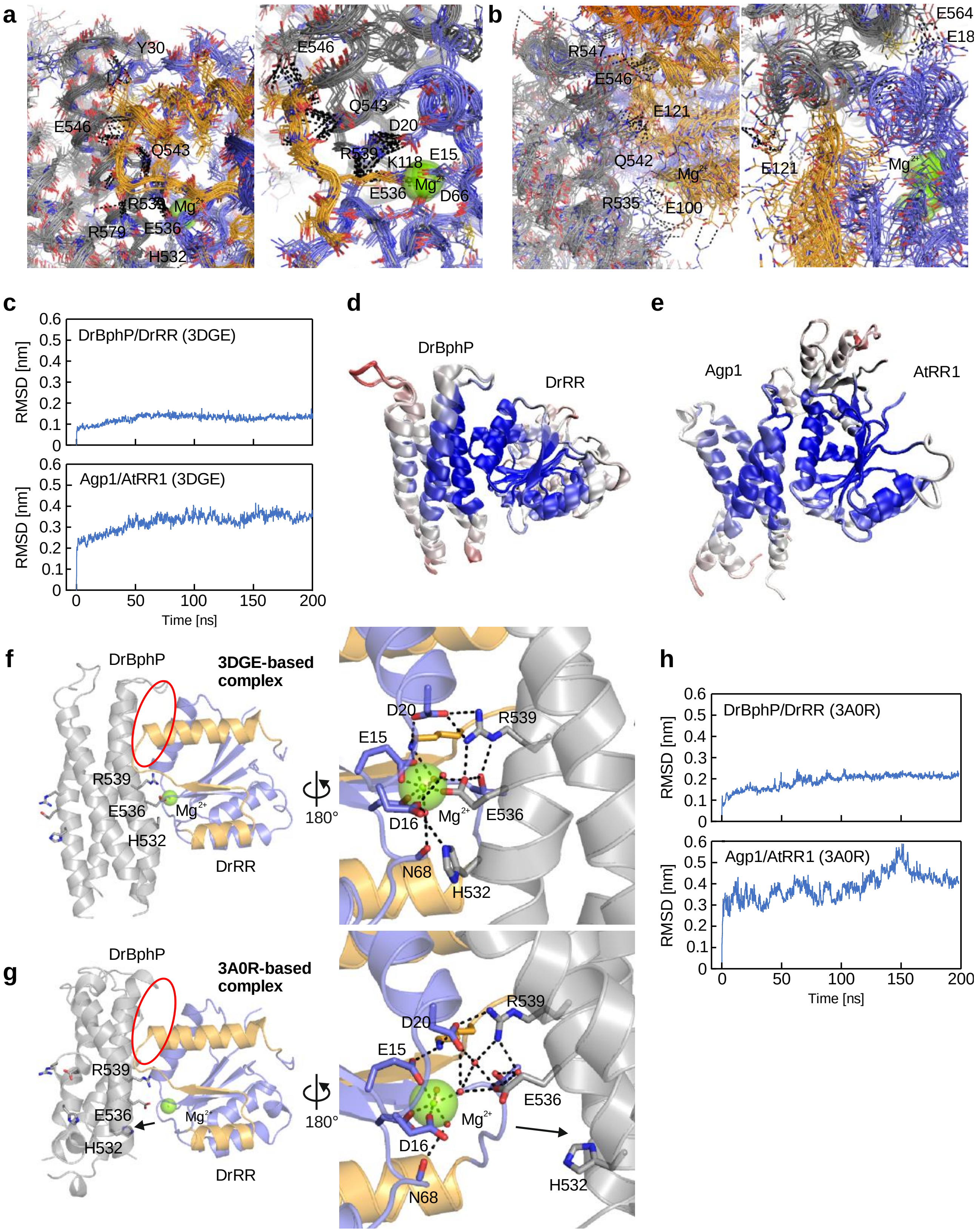


**Supplementary Figure 5.** Flexibility of the HK/RR complexes in Agp1 and DrBphP. **a–b**. Relaxed solvated models of complexes DrBphP/DrRR (a) and Agp1/AtRR1 (b). 11 representative models are shown after global structural alignment by using PyMOL Molecular Graphics System version 2.3.3 (Schrödinger, LLC). Some of the polar interactions and salt bridges have been indicated. Molecules have been coloured as in Figure 5 of the

main text. **c**. RMSD of the backbone atoms along the 200 ns MD trajectory of DrBphP/DrRR complex and Agp1/AtRR1 complex made homologically from the 3DGE structure [4]. The RMSD values are relative to the starting structure, and the stability of the complex is reflected by the fluctuation of RMSD values. **d–e**. Colour maps of the amino acids heavy atoms root-mean square fluctuations (RMSF) for DrBphP/DrRR complex (d) and Agp1/AtRR1 complex (e). Blue colour denotes rigid regions, red colour denotes more flexible regions. **f–g**. Comparison of the DrBphP/DrRR complexes achieved from different starting models. In panel f, the complex is based on 3DGE [4] model and shown in Figure 5. In panel g, the starting model is based on crystal structure 3A0R [5]. Although the main interaction interface, denoted as red circle, is similar between the models, the bending of the DHp helix inherited from the 3A0R complex structure (g) reduces the amount of E536 contacts with DrRR. Due to the DHp bending, H532 resides far from the DrRR active site, but the R539 retains its interactions. It is possible that the 3A0R complex models represents a different HK activity state or the bending of the DHp helices result from crystallization artifacts [4]. **h**. RMSD of the backbone atoms along the 200 ns MD trajectory of DrBphP/DrRR complex and Agp1/AtRR1 complex made homologically from the 3A0R structure [5]. Note that the DrBphP/DrRR complex appears slightly less stable than in panel c (more fluctuation of the RMSD values). Agp1/AtRR1 complex was highly unstable and deemed to dissociate during simulation.

**Supplementary Figure 6**

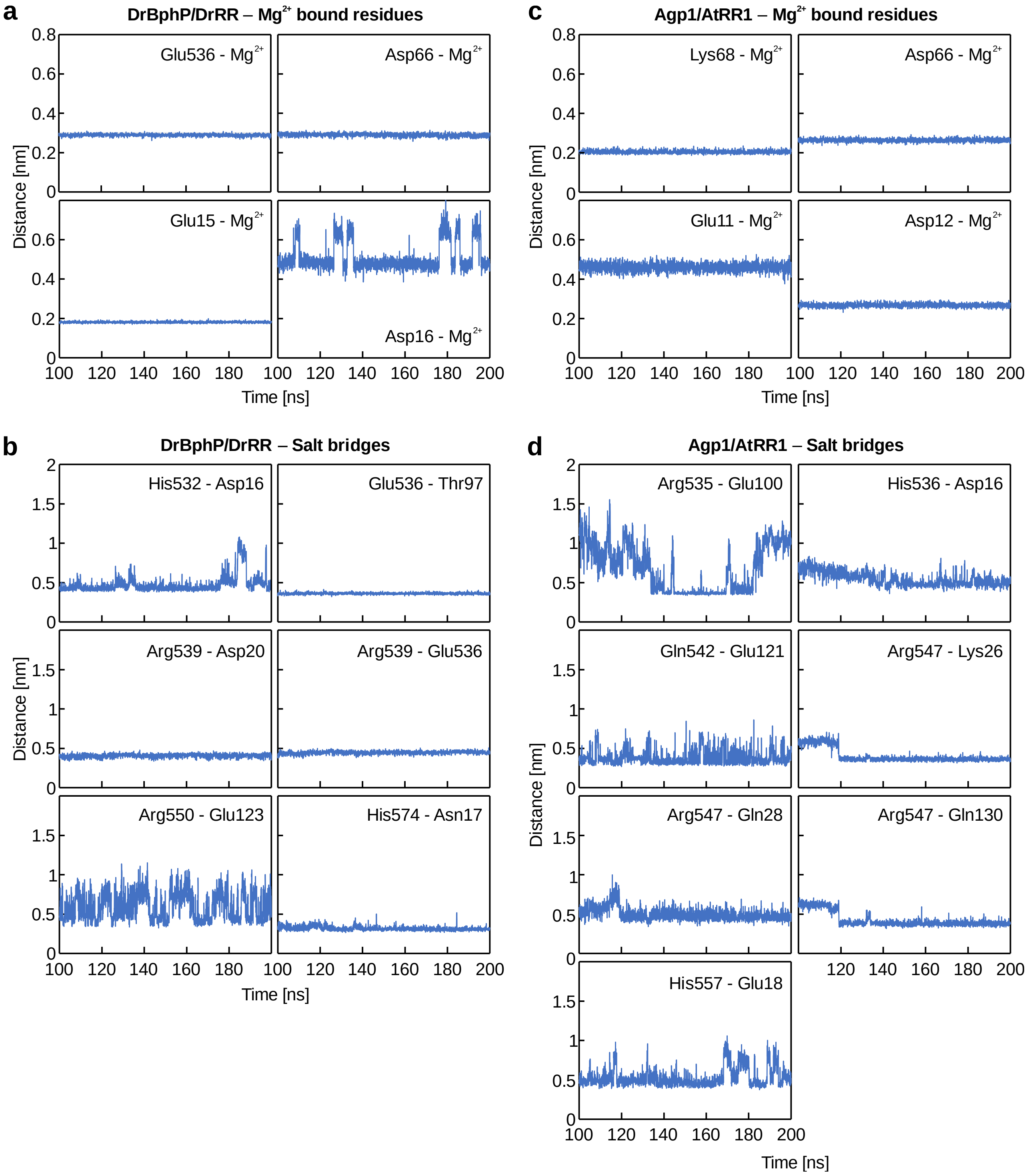


**Supplementary Figure 6.** Distance plots of the structural parameters in HK/RR complexes. Each individual plot represents a distance between the head groups of the residues at 100–200 ns in the MD trajectory after the initial 100 ns equilibration. **a**. Distances between $Mg^{2+}$ ion and head groups of bound residues in DrBphP/DrRR complex. **b**. Salt bridges between DrRR and DrBphP proteins. **c**. distances between $Mg^{2+}$ ion and head groups of bound residues in Agp1/AtRR1 complex. **d**. Salt bridges between AtRR1 and Agp1 proteins.

## Supplementary Figure 7

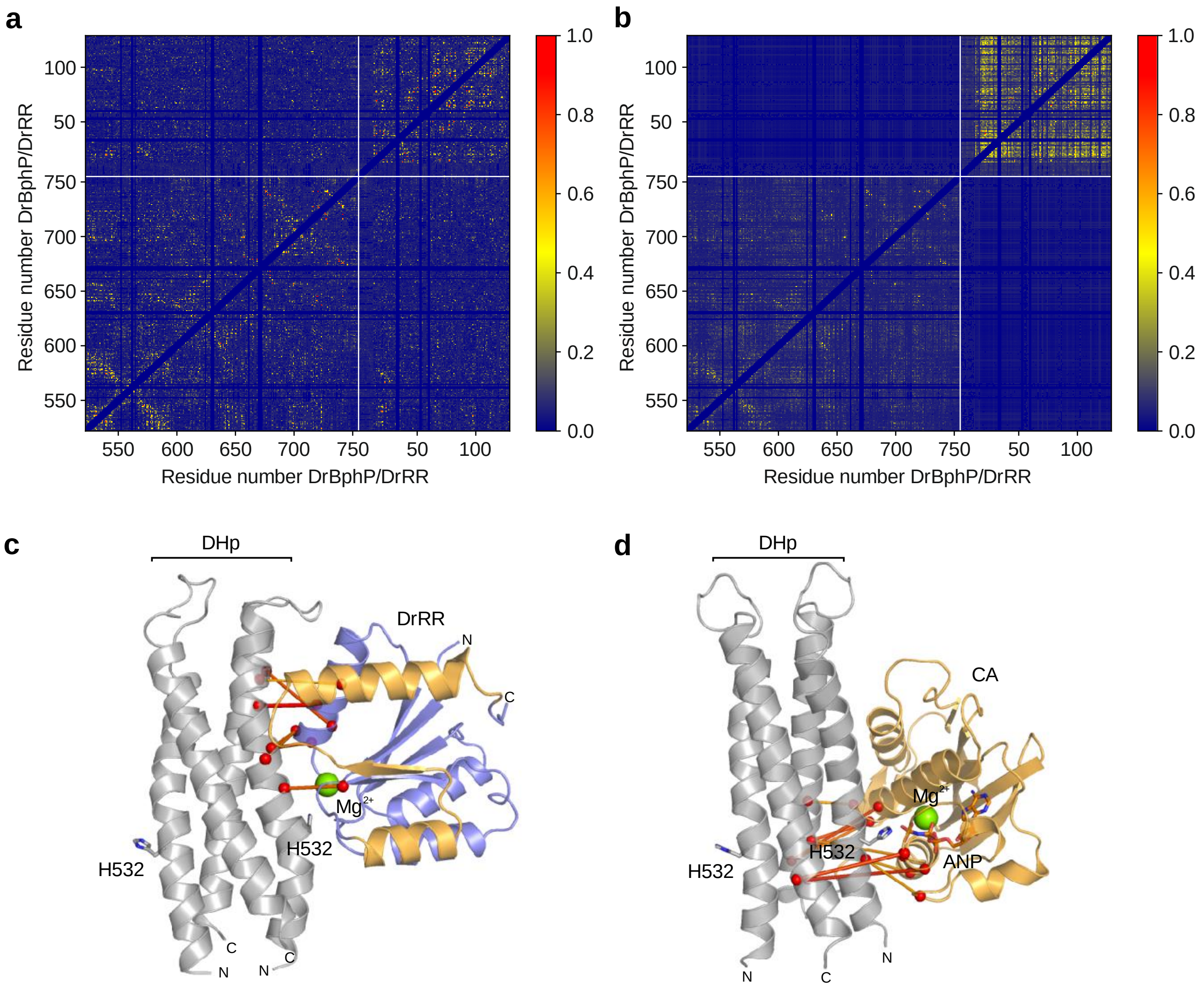


**Supplementary Figure 7.** Covariance analysis of in the DHp/RR and DHp/CA interfaces. **a**. Covariance matrix of the tandem BphP-RR sequences. Blue colour indicates low and red high pairwise covariance. **b**. Covariance matrix of scrambled sequences. Here, little covariance between the HK and RR sequence segments is visible, thus verifying the specificity of the results shown in panel a. **c–d**. Strong covariances between residues in the DHp helices and their cognate response regulators (RR), and a catalytic ATP-binding (CA) domains. The same models are shown as in Figures 5a and 6a. The extent of the covariance is colour-coded in such a way that the red lines indicate high covariance and yellow lines intermediate covariance. Only the covariance lines above a cutoff value of 0.6 are shown.

## Supplementary Figure 8

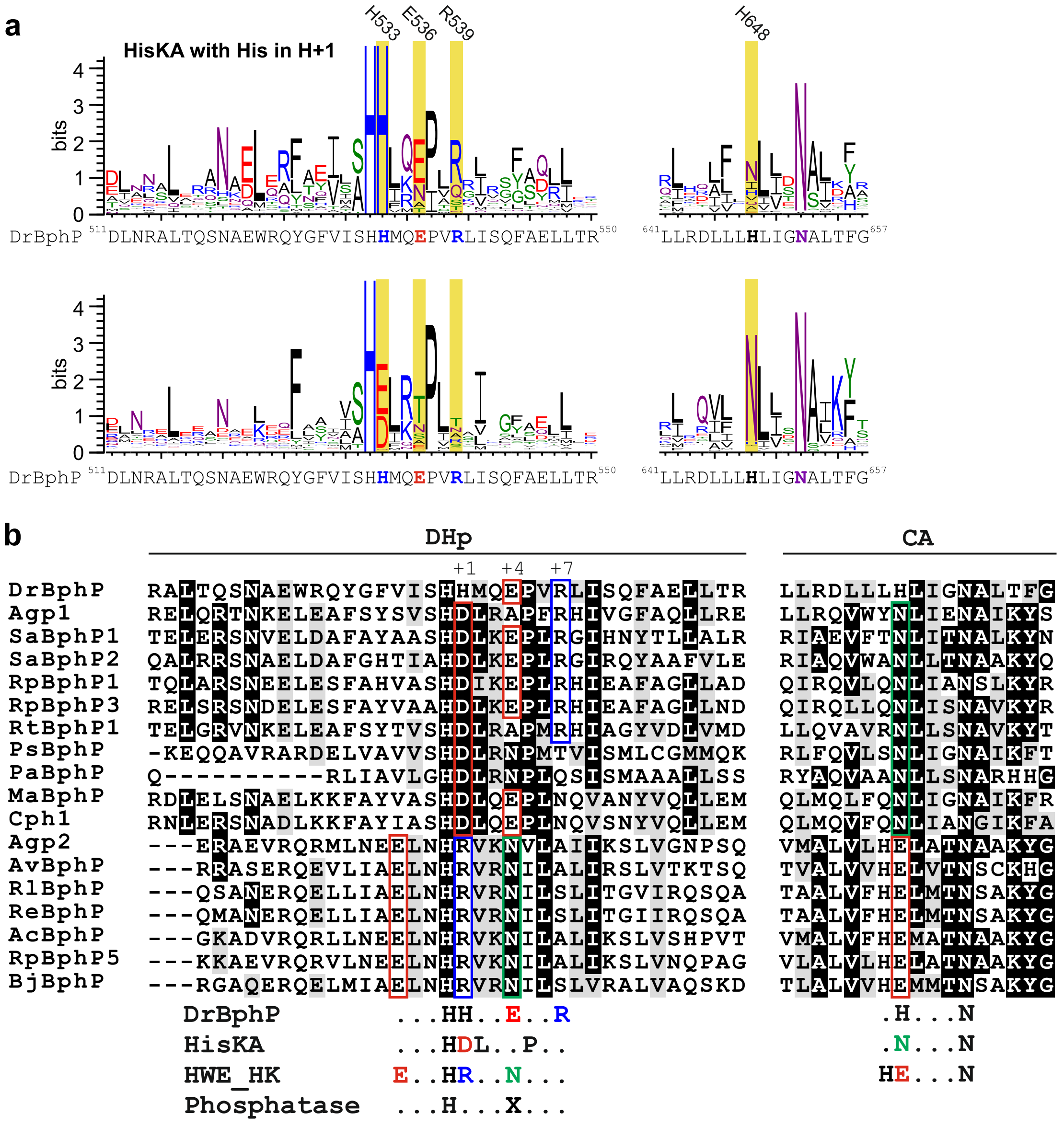

**Supplementary Figure 8.** Sequence variation of the key catalytic residues in histidine kinases. **a**. Sequence logo showing the conservation of selected residues in the HisKA occurrences. Two occasions have been shown: One where the H+1 position in the DHp helix is occupied by a histidine (His533 in DrBphP), another where H+1 site is not restricted to as a histidine. The sequence logo in the former case were plotted from 437 sequences, which resulted in a noisier graph than in the latter case. In the case where H+1 is histidine, the conservation of the N-box Asn in the CA domain is lost (H648 in DrBphP). However, the occurrence of Glu in H+4 position as well as Arg in position H+7 is increased in these cases. The key residues are shaded in yellow. **b**. Sequence alignment of representative bacteriophytochromes. Below the alignment, the key DrBphP residues discussed in the main text are shown, along with consensus sequence of HisKA, HWE_HK, and phosphatase. The alignment shows that the bacteriophytochromes above Cph1, and Cph1 itself, likely belong to HisKA family. Although DrBphP resembles other HisKA proteins, it misses a few functionally important residues. Agp1 has all the key residues required for HisKA activity. Agp2 and the phytochromes below it belong to HWE_HK proteins with a characteristic arginine residue in its H+1 position and a glutamate in the N-box of the CA domain. As for phosphatase activity, a threonine, asparagine or glutamate in H+4 position indicate that all selected phytochromes may also act as phosphatases. As an exception, the phosphatase activity of Agp1 may be hindered due to an alanine in this position. Also RtBphP1 has an alanine in the H+4 position, which hints for functional resemblance to Agp1. This is supported by their similar cognate response regulator

that form arm-in-arm dimers [6]. In conclusion, most phytochrome sequences have features that enable both kinase and phosphatase activity. Notable exceptions for this are Agp1 and DrBphP. Full-length sequences were aligned in Jalview 2.11.1.0 [7] by using ClustalO version 1.2. [8,9] with standard settings. The amino acid conservation was visualized with Boxshade version 3.21. Uniprot accession numbers in the same order as in the alignment: DrBphP (*Deinococcus radiodurans* - Q9RZA4), Agp1 (*Agrobacterium fabrum* - Q7CY45), SaBphP1 (*Stigmatella aurantiaca* - Q097N3), SaBphP2 (*Stigmatella aurantiaca* - Q09E27), RpBphP1 (*Rhodopseudomonas palustris* - Q6N5G3), RpBphP3 (*Rhodopseudomonas palustris* - Q6N5G2), RtBphP1 (*Ramlibacter tataouinensis* - F5Y2U7), PsBphP (*Pseudomonas syringae* - Q885D3), PaBphP (*Pseudomonas aeruginosa* - Q9HWR3), MaBphP (*Microcystis aeruginosa* - B0JT05), Cph1 (*Synechocystis sp. PCC 6803* - Q55168), Agp2 (*Agrobacterium fabrum* - A9CI81), AvBphP (*Agrobacterium vitis* - B9K3G4), RlBphP (*Rhizobium leguminosarum* - Q1MCX7), ReBphP (*Rhizobium etli* - B3PX96), AcBphP (*Azorhizobium caulinodans* - A8HU76), RpBphP5 (*Rhodopseudomonas palustris* - Q6NB40), BjBphP (*Bradyrhizobium japonicum* - A0A023X9Y5).

**Supplementary Figure 9**

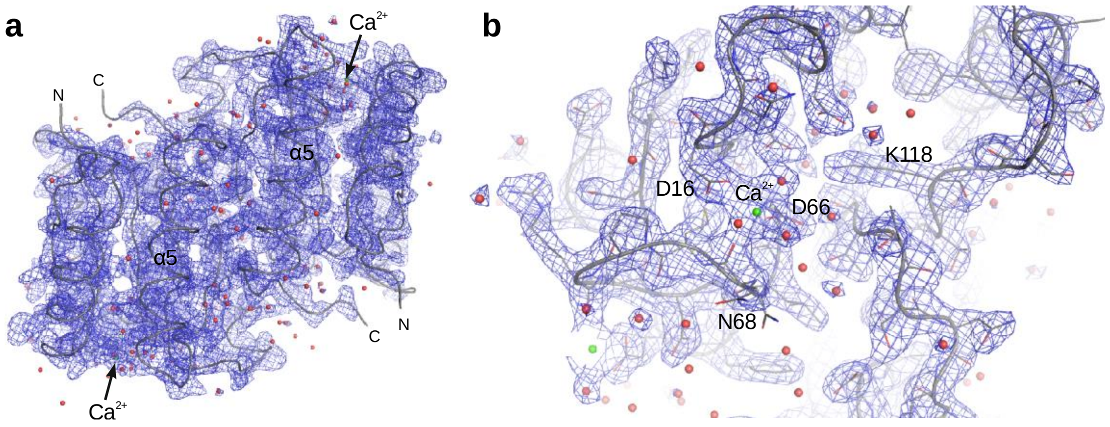


**Supplementary Figure 9**. Electron density of the DrRR crystal structure. **a**. One of the DrRR dimers of the asymmetric unit constituting of A and B chains. The orientation of the dimer is similar to Figure 4a of the main text. **b**. Active site of chain A with a bound $Ca^{2+}$ ion. The $2F_o$–$F_c$ maps are shown as blue mesh at 1.5 σ, and calculated with programs Fft and Mapmask of the CCP4 Interface version 7.0.078 [10]. Figures were generated with PyMOL Molecular Graphics System version 2.3.3 (Schrödinger, LLC).

**Supplementary Table 1**

| Primer name | Sequence (5'→3') | Use of the primer |
|---|---|---|
| DrBphP_H533D | ttcgttatcagccacgacatgcaggagccggtg | DrBphP mutagenesis [a] |
| DrBphP_E536A | agccaccacatgcaggcgccggtgcggctcatc | |
| DrBphP_E536T | agccaccacatgcagacgccggtgcggctcatc | |
| DrBphP_E536D | agccaccacatgcaggacccggtgcggctcatc | |
| DrBphP_E536Q | agccaccacatgcagcaaccggtgcggctcatc | |
| DrBphP_E536N | agccaccacatgcagaacccggtgcggctcatc | |
| DrBphP_R539A | atgcaggagccggtggcgctcatctcgcagttcg | |
| Agp1_D529H | cctattccgtttcgcatcacctgcgcgcgccgttcc | Agp1 mutagenesis [a] |
| AgP1_A532E | tcgcatgacctgcgcgaaccgttccgtcatattgtc | |
| DrBphP_512_XhoI | gagcgtgattcgtgacctcctcgaggcgctcacacagtcg | Chimera cloning [b] |
| Agp1_XhoI_511 | tatatatatatactcgaggagttgcagcgcaccaataaggag | |
| Agp1_745_XhoI_rev | tatatatatatactcgagggcaattttttcctcttcaactttcg | |
| FL_AtHK_XhoI_remove | agcgtgattcgtgacctcacacgggagttgcagcgcaccaataag | |
| DrRR_XhoI_1 | atatatatatctcgagctatgcctgagcgcgcctcc | DrRR to pET21b [b] |
| DrRR_149_BamHI_rev | atatatataggatccctgcggctggtacgtctgc | |
| AtRR_BamHI_1 | tattatataggatccatgcctgaactcagacccattc | AtRR1 to pET21b [b] |
| AtRR_149_XhoI_rev | atatatatactcgagtccgccattgcgatatgc | |
| ArRR_linker_for | GCTGGTTCTGCCGGCTCCGCTGGTatgcctgaactcag acccattctgc | EGFP-RR cloning [c] |
| DrRR_linker_for | GCTGGTTCTGCCGGCTCCGCTGGTatgcctgagcgcgc ctc | |
| EGFP_linker_rev | GAGCCGGCAGAACCAGCACTATCCTTGTACAGCTC GTCCATGCCG | |

[a] Mutated nucleotides underlined
[b] Restriction sites underlined
[c] Linker sequence underlined

**Supplementary Table 1**. DNA primers used in this study. The mutated codons, introduced restriction sites, and linker sequences are underlined.

## Supplementary References


1 Bai, Y., Rottwinkel, G., Feng, J., Liu, Y. & Lamparter, T. Bacteriophytochromes control conjugation in Agrobacterium fabrum. *J. Photochem. Photobiol. B.* **161**, 192-199, doi:10.1016/j.jphotobiol.2016.05.014 (2016).

2 Takala, H., Lehtivuori, H., Hammaren, H., Hytonen, V. P. & Ihalainen, J. A. Connection between absorption properties and conformational changes in Deinococcus radiodurans phytochrome. *Biochemistry* **53**, 7076-7085, doi:10.1021/bi501180s (2014).

3 Takala, H., Björling, A., Linna, M., Westenhoff, S. & Ihalainen, J. A. Light-induced Changes in the Dimerization Interface of Bacteriophytochromes. *J. Biol. Chem.* **290**, 16383-16392, doi:10.1074/jbc.M115.650127 (2015).

4 Casino, P., Rubio, V. & Marina, A. Structural insight into partner specificity and phosphoryl transfer in two-component signal transduction. *Cell* **139**, 325-336, doi:10.1016/j.cell.2009.08.032 (2009).

5 Yamada, S. *et al.* Structure of PAS-linked histidine kinase and the response regulator complex. *Structure* **17**, 1333-1344, doi:10.1016/j.str.2009.07.016 (2009).

6 Baker, A., Satyshur, K., Moreno Morales, N. & Forest, K. Arm-in-Arm Response Regulator Dimers Promote Intermolecular Signal Transduction. *J. Bacteriol.* **198**, 1218-1229, doi:10.1128/JB.00872-15 (2016).

7 Waterhouse, A. M., Procter, J. B., Martin, D. M., Clamp, M. & Barton, G. J. Jalview Version 2--a multiple sequence alignment editor and analysis workbench. *Bioinformatics* **25**, 1189-1191, doi:10.1093/bioinformatics/btp033 (2009).

8 Sievers, F. & Higgins, D. G. Clustal Omega for making accurate alignments of many protein sequences. *Protein Sci.* **27**, 135-145, doi:10.1002/pro.3290 (2018).

9 Sievers, F. *et al.* Fast, scalable generation of high-quality protein multiple sequence alignments using Clustal Omega. *Mol. Syst. Biol.* **7**, 539, doi:10.1038/msb.2011.75 (2011).

10 Potterton, E., Briggs, P., Turkenburg, M. & Dodson, E. A graphical user interface to the CCP4 program suite. *Acta Crystallogr. D Biol. Crystallogr.* **59**, 1131-1137, doi:10.1107/s0907444903008126 (2003).
