# Supplementary material for: Comparative analysis of two paradigm bacteriophytochromes reveals opposite functionalities in two-component signaling
Source: Nat Commun. 2021 Jul 20;12:4394. doi: 10.1038/s41467-021-24676-7 (PMC8292422; doi:10.1038/s41467-021-24676-7)
Supplement: Supplementary file 3 — Description of Additional Supplementary Files [file 41467_2021_24676_MOESM3_ESM.docx]

**Description of Additional Supplementary Files**

Description: tabulated PSICOV scores

File Name: Supplementary Data 1
