# Supplementary material for: Comparative analysis of two paradigm bacteriophytochromes reveals opposite functionalities in two-component signaling
Source: Nat Commun. 2021 Jul 20;12:4394. doi: 10.1038/s41467-021-24676-7 (PMC8292422; doi:10.1038/s41467-021-24676-7)
Supplement: Supplementary file 5 — Reporting Summary [file 41467_2021_24676_MOESM5_ESM.pdf]

nature research

Corresponding author(s): Dr. Heikki Takala
Prof. Janne A. Ihalainen

Last updated by author(s): Jun 29, 2021

Reporting Summary

Nature Research wishes to improve the reproducibility of the work that we publish. This form provides structure for consistency and transparency in reporting. For further information on Nature Research policies, see our Editorial Policies and the Editorial Policy Checklist.

Statistics

For all statistical analyses, confirm that the following items are present in the figure legend, table legend, main text, or Methods section.

n/a	Confirmed	
[x]	[ ]	The exact sample size (*n*) for each experimental group/condition, given as a discrete number and unit of measurement
[ ]	[x]	A statement on whether measurements were taken from distinct samples or whether the same sample was measured repeatedly
[x]	[ ]	The statistical test(s) used AND whether they are one- or two-sided<br>*Only common tests should be described solely by name; describe more complex techniques in the Methods section.*
[x]	[ ]	A description of all covariates tested
[x]	[ ]	A description of any assumptions or corrections, such as tests of normality and adjustment for multiple comparisons
[ ]	[x]	A full description of the statistical parameters including central tendency (e.g. means) or other basic estimates (e.g. regression coefficient) AND variation (e.g. standard deviation) or associated estimates of uncertainty (e.g. confidence intervals)
[x]	[ ]	For null hypothesis testing, the test statistic (e.g. *F*, *t*, *r*) with confidence intervals, effect sizes, degrees of freedom and *P* value noted<br>*Give P values as exact values whenever suitable.*
[x]	[ ]	For Bayesian analysis, information on the choice of priors and Markov chain Monte Carlo settings
[x]	[ ]	For hierarchical and complex designs, identification of the appropriate level for tests and full reporting of outcomes
[x]	[ ]	Estimates of effect sizes (e.g. Cohen's *d*, Pearson's *r*), indicating how they were calculated

Our web collection on statistics for biologists contains articles on many of the points above.

Software and code

Policy information about availability of computer code

Data collection	Crystallography data were collected at ESRF ID23-2 by using its own software. Sequences were retrieved from GenBank with Biopython ver. 1.77 and were analyzed with custom Python (ver. 3.6.3) scripts.
Data analysis	Crystal data was processed and structure generated with XDS package (January 26, 2018), Phaser (ver. 2.5.7), REFMAC (ver. 5.8.0135), and Coot 0.8.2. Sequences were aligned and clustered with muscle (ver. 3.8.31) and usearch (ver. 11.0.667) and uclust (ver. 11.0.667); covariance analysis was carried out with psicov ver. 1.10D; sequence logos were generated with WebLogo ver. 3.6.0. for sequence analysis BLASTP (ver. 2.10.0) was used. MD simulations were done with Gromacs 2018.8. package. Structure figures were generated with PyMOL Molecular Graphics System version 2.3.3

For manuscripts utilizing custom algorithms or software that are central to the research but not yet described in published literature, software must be made available to editors and reviewers. We strongly encourage code deposition in a community repository (e.g. GitHub). See the Nature Research guidelines for submitting code & software for further information.

Data

Policy information about availability of data

All manuscripts must include a data availability statement. This statement should provide the following information, where applicable:

- Accession codes, unique identifiers, or web links for publicly available datasets
- A list of figures that have associated raw data
- A description of any restrictions on data availability

Crystallography data that support the findings of this study have been deposited in The Worldwide Protein Data Bank archive (http://www.wwpdb.org/) with an accession code 6XVU (https://doi.org/10.2210/pdb6XVU/pdb). The models generated and analyzed simulation parameters and force fields used in the current study are available in the GitHub repository (https://github.com/dmmorozo/HK-RR-simulations) with DOI (https://doi.org/10.5281/zenodo.4922582). The sequence

datasets analyzed within this study are available in Zenodo repository unde DOI (https://doi.org/10.5281/zenodo.5005587). Database entries used in this study are listed in the Data Availability section. The authors declare that all relevant data supporting the findings of this study will available within the paper and its supplementary information files. Raw data and full gels associated to Figure 1-3 and Supporting Figures 1-4 are included as separate Source Data files. The Source Data files include representative versions of the gels, and the remaining gels that cover the replicate experiments are available upon request.

Field-specific reporting

Please select the one below that is the best fit for your research. If you are not sure, read the appropriate sections before making your selection.

- [x] Life sciences
- [ ] Behavioural & social sciences
- [ ] Ecological, evolutionary & environmental sciences

For a reference copy of the document with all sections, see nature.com/documents/nr-reporting-summary-flat.pdf

Life sciences study design

All studies must disclose on these points even when the disclosure is negative.

Sample size	Each reported measurement was repeated independently at least three times. The amount of repeats or data points is defined in each reported analysis.
Data exclusions	In Surface Plasmon Resonance the sharp peaks corresponding to the injection start (0 s) and stop (120 s) in each sensorgram were excluded from the analysis. In isothermal Titration Calorimetry, a few outlier peaks were excluded from the analyses. Exclusion criteria were pre-established. Otherwise, no data was excluded from the measurements.
Replication	All attempts at replication were successful. The experimenst were repeated at least three times independently.
Randomization	Randomization was not employed.
Blinding	Blinding was not employed.

Reporting for specific materials, systems and methods

We require information from authors about some types of materials, experimental systems and methods used in many studies. Here, indicate whether each material, system or method listed is relevant to your study. If you are not sure if a list item applies to your research, read the appropriate section before selecting a response.

Materials & experimental systems

n/a	Involved in the study	
x		Antibodies
x		Eukaryotic cell lines
x		Palaeontology and archaeology
x		Animals and other organisms
x		Human research participants
x		Clinical data
x		Dual use research of concern

Methods

n/a	Involved in the study	
x		ChIP-seq
x		Flow cytometry
x		MRI-based neuroimaging
