# Supplementary figures and images for: Comparative analysis of two paradigm bacteriophytochromes reveals opposite functionalities in two-component signaling
Source: Nat Commun. 2021 Jul 20;12:4394. doi: 10.1038/s41467-021-24676-7 (PMC8292422; doi:10.1038/s41467-021-24676-7)

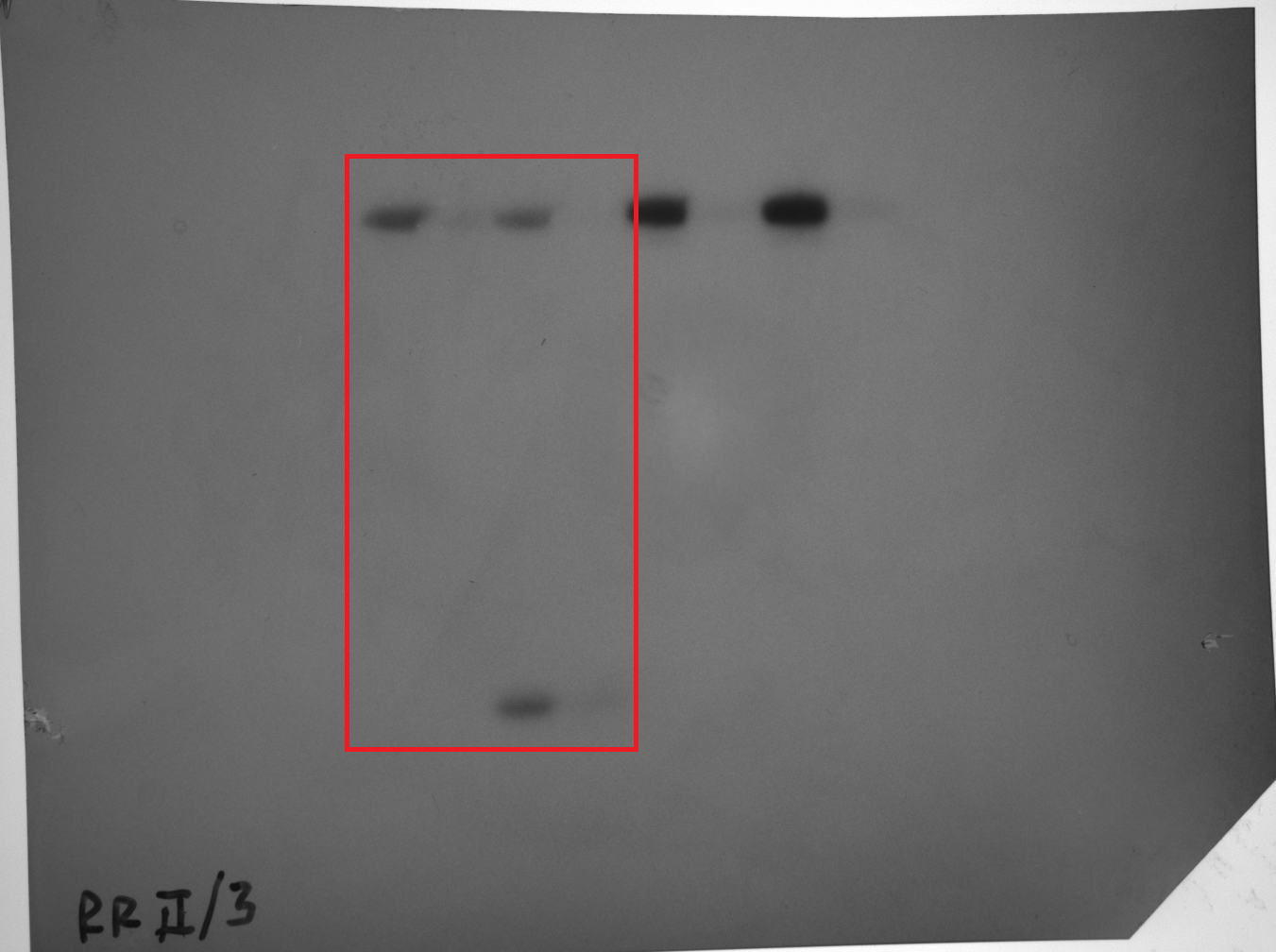

Supplement: Supplementary file 6 — Source Data [file 41467_2021_24676_MOESM6_ESM.zip › Figure 3 gels/3A_Agp1_phosphate.tif]

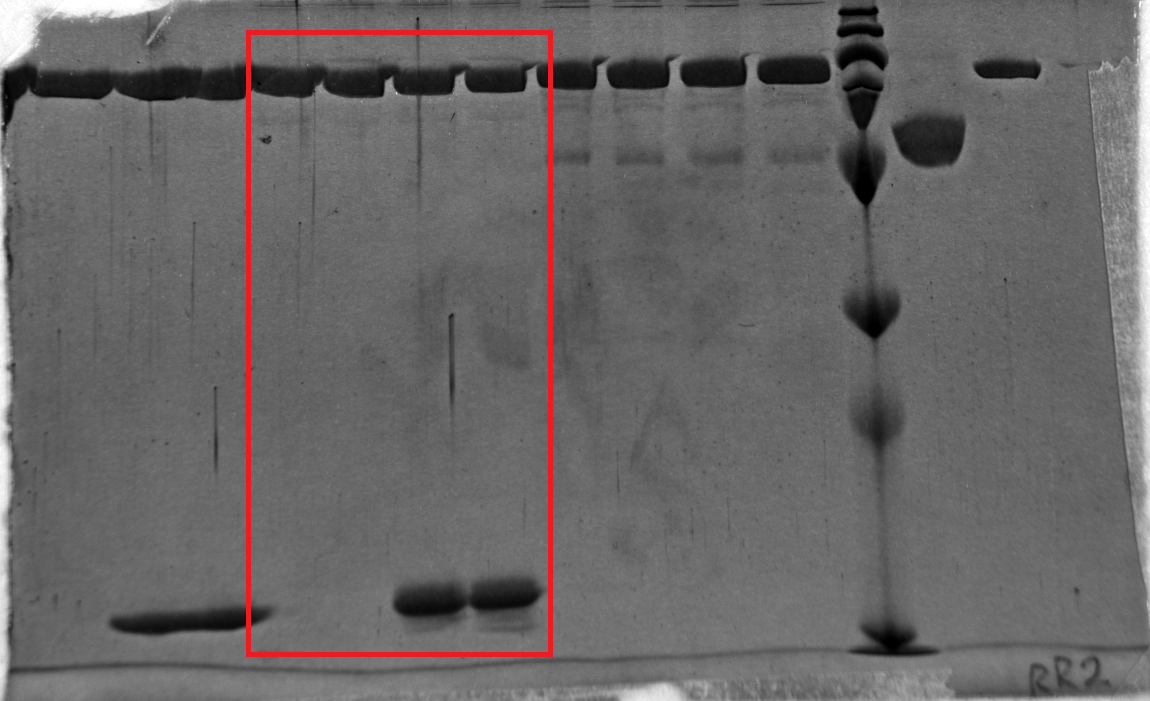

Supplement: Supplementary file 6 — Source Data [file 41467_2021_24676_MOESM6_ESM.zip › Figure 3 gels/3A_Agp1_protein.tif]

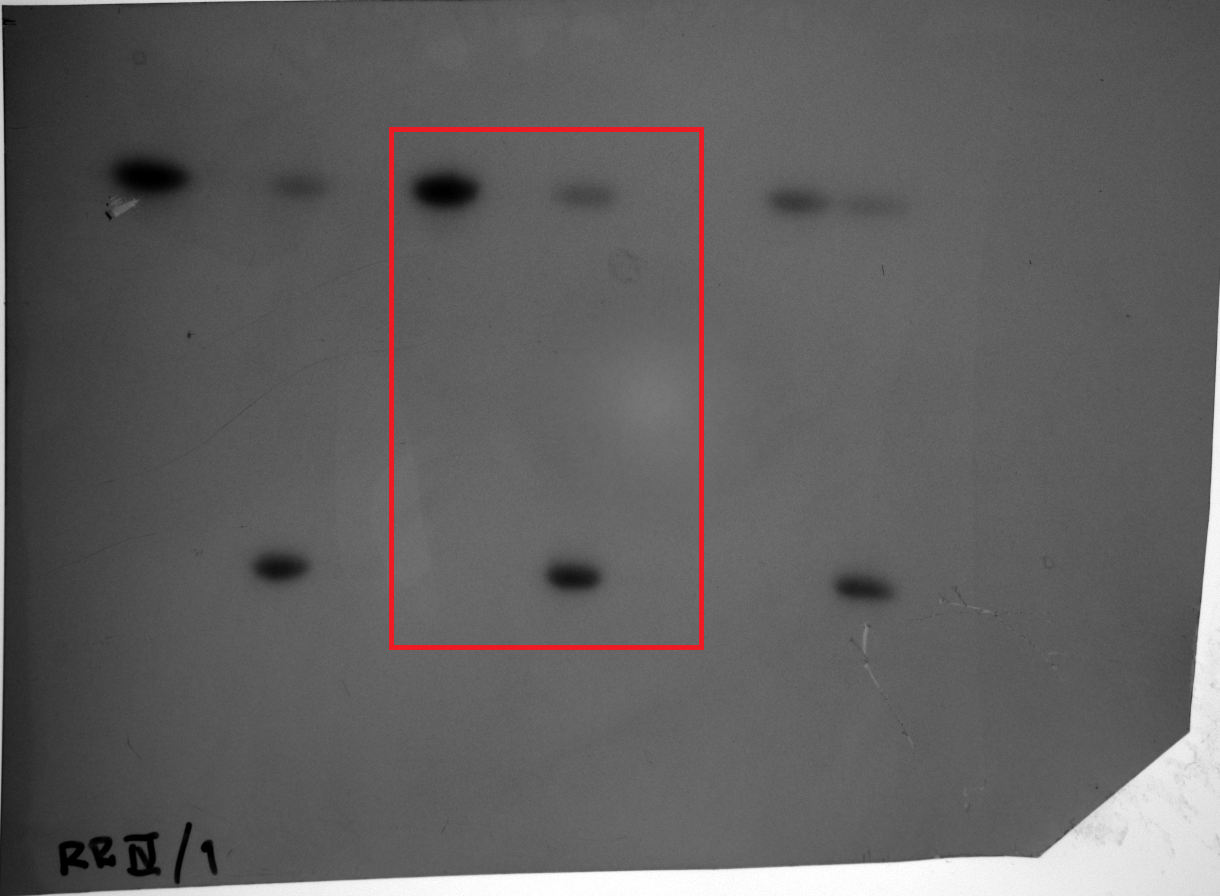

Supplement: Supplementary file 6 — Source Data [file 41467_2021_24676_MOESM6_ESM.zip › Figure 3 gels/3A_Chimera_Phosphate.tif]

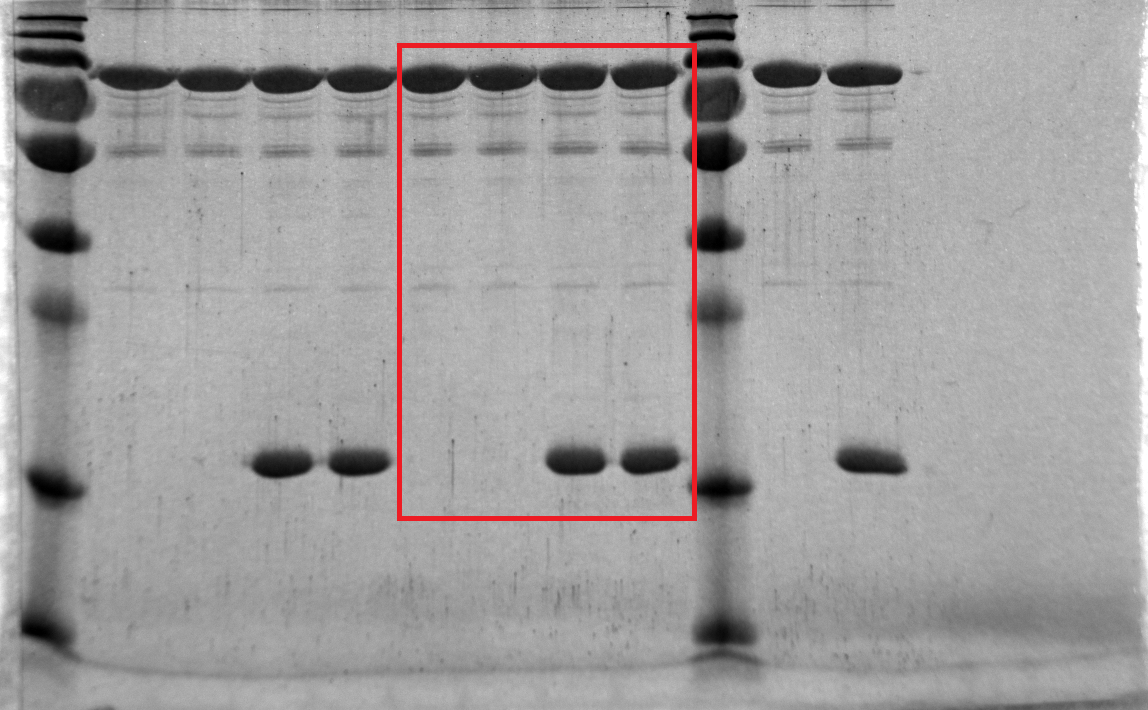

Supplement: Supplementary file 6 — Source Data [file 41467_2021_24676_MOESM6_ESM.zip › Figure 3 gels/3A_Chimera_protein.tif]

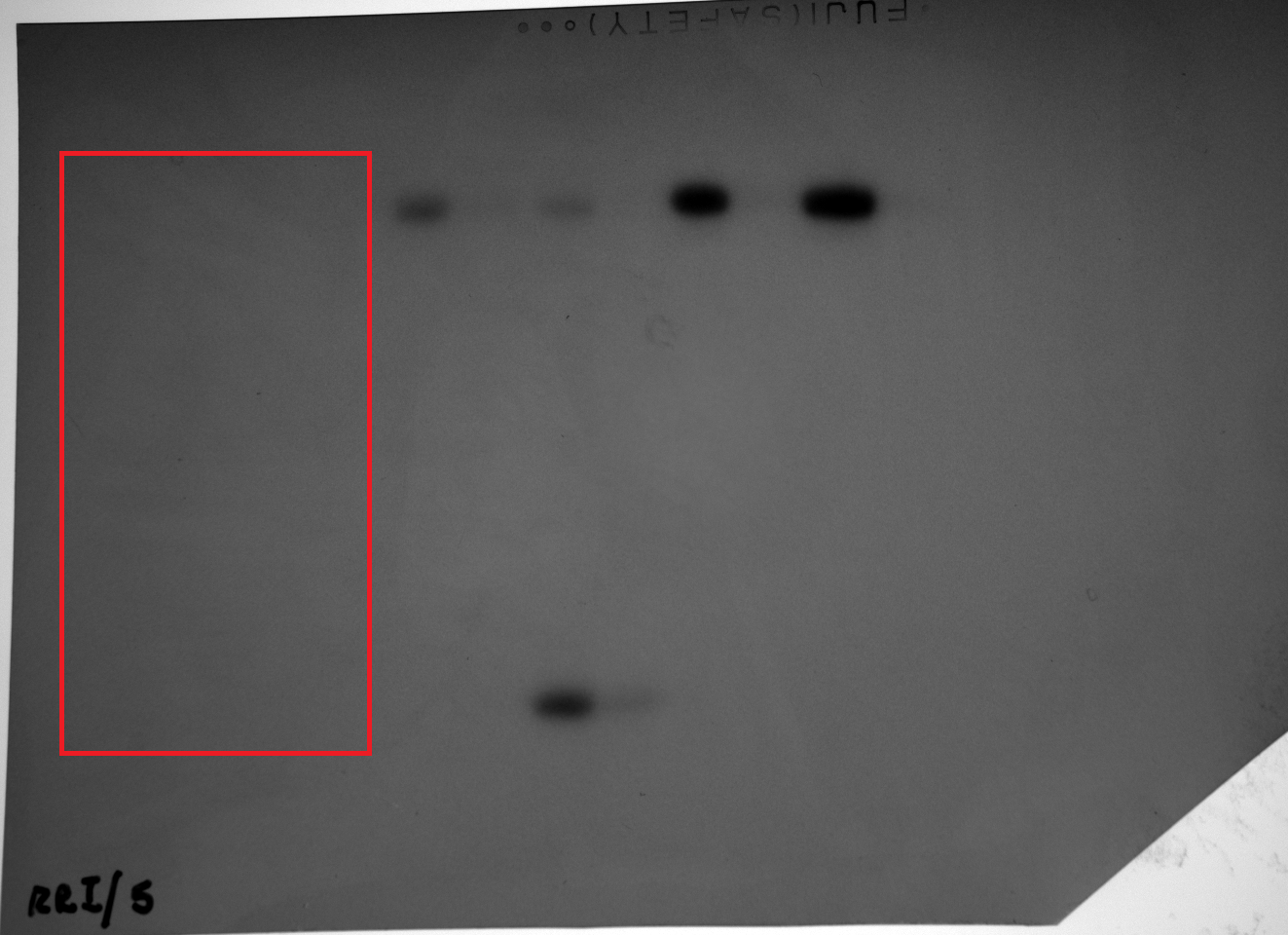

Supplement: Supplementary file 6 — Source Data [file 41467_2021_24676_MOESM6_ESM.zip › Figure 3 gels/3A_DrBphP_phosphate.tif]

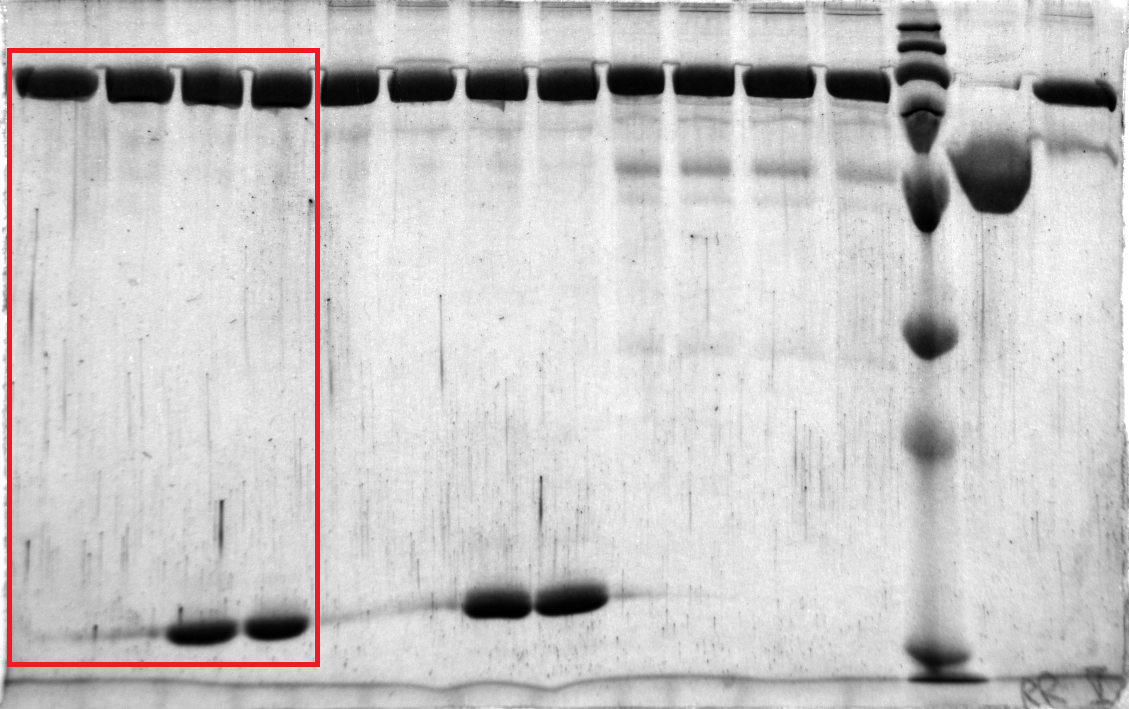

Supplement: Supplementary file 6 — Source Data [file 41467_2021_24676_MOESM6_ESM.zip › Figure 3 gels/3A_DrBphP_protein.tif]

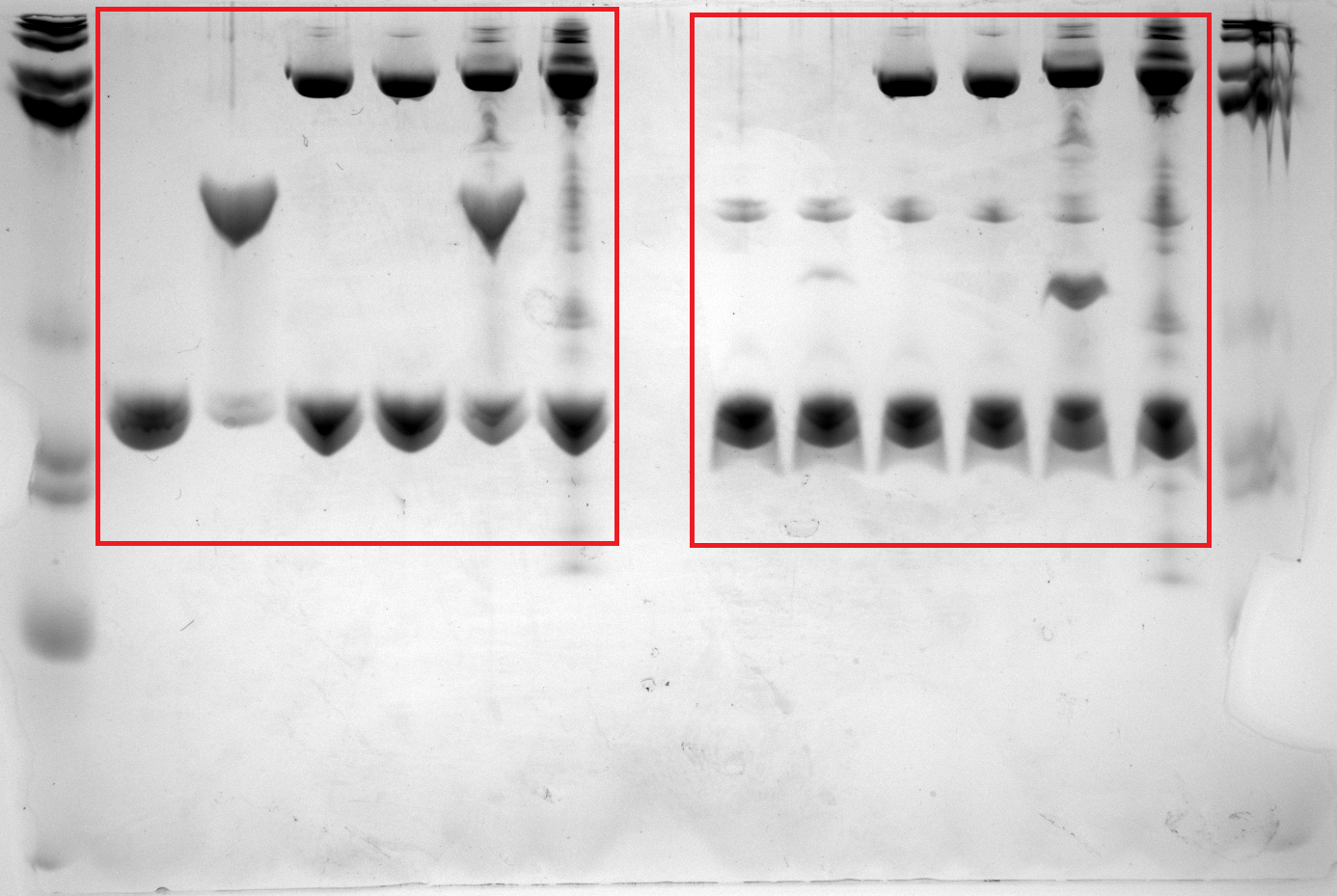

Supplement: Supplementary file 6 — Source Data [file 41467_2021_24676_MOESM6_ESM.zip › Figure 3 gels/3B.tif]

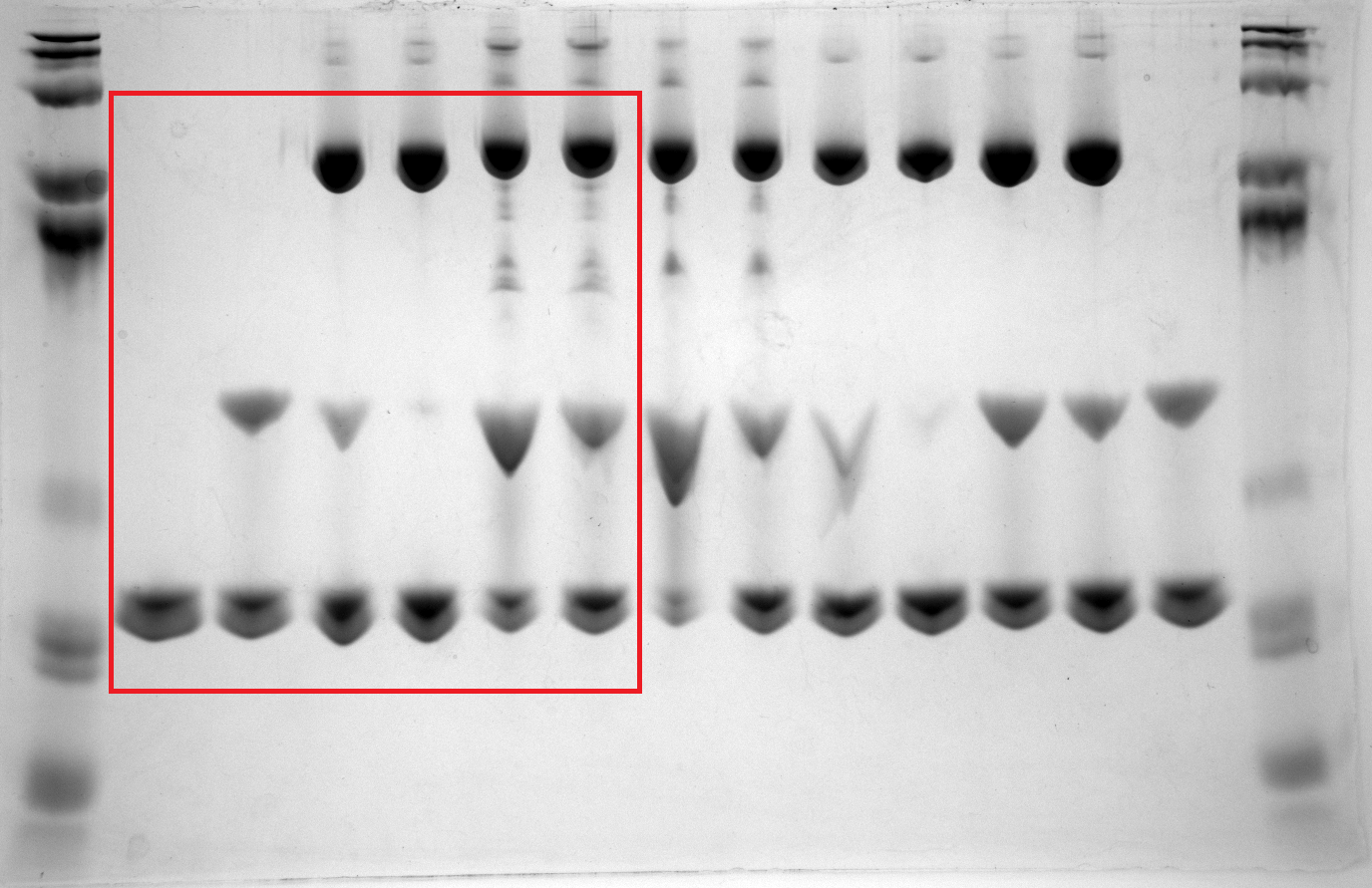

Supplement: Supplementary file 6 — Source Data [file 41467_2021_24676_MOESM6_ESM.zip › Figure 3 gels/3C.tif]

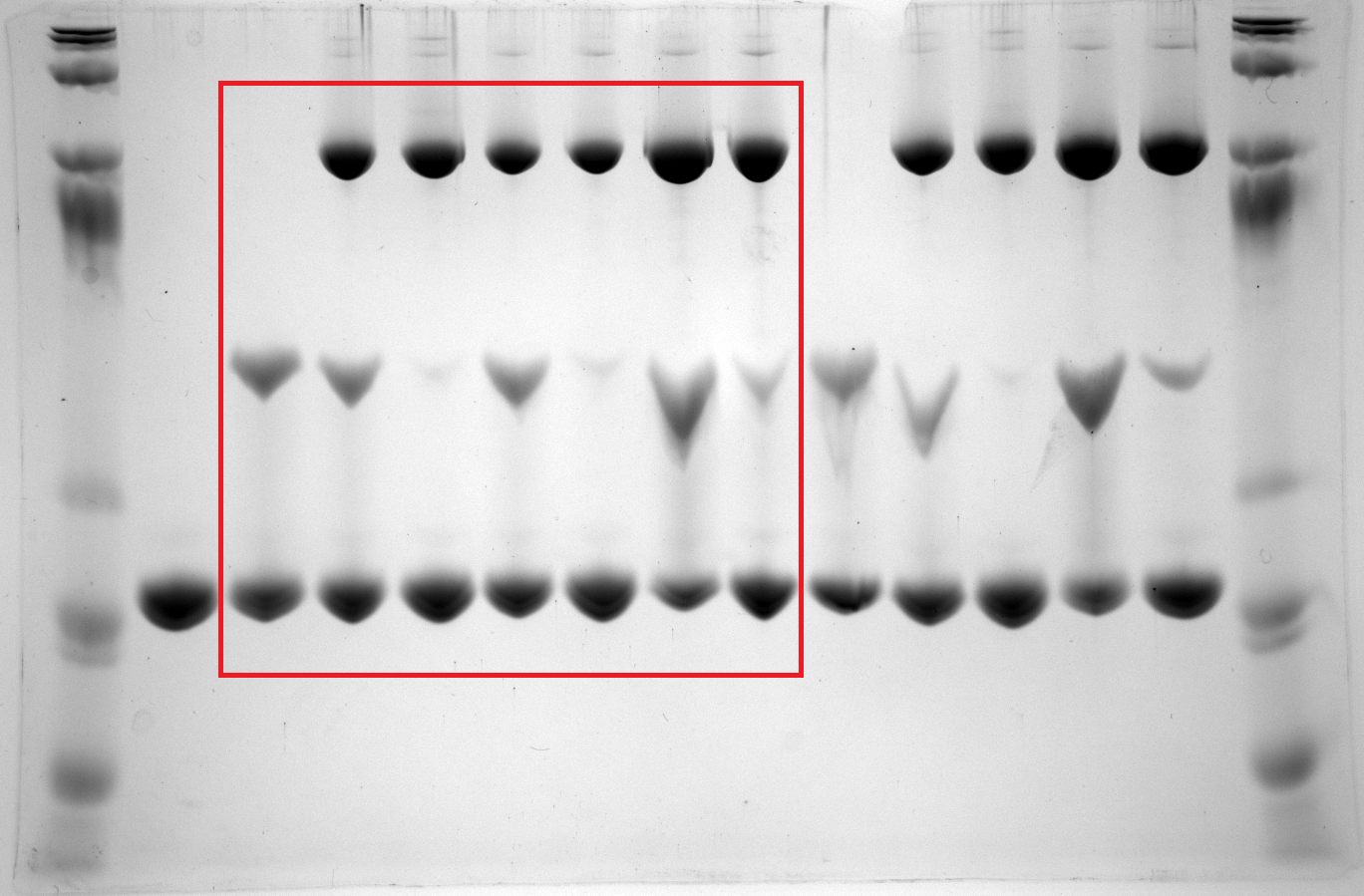

Supplement: Supplementary file 6 — Source Data [file 41467_2021_24676_MOESM6_ESM.zip › Figure 3 gels/3D.tif]

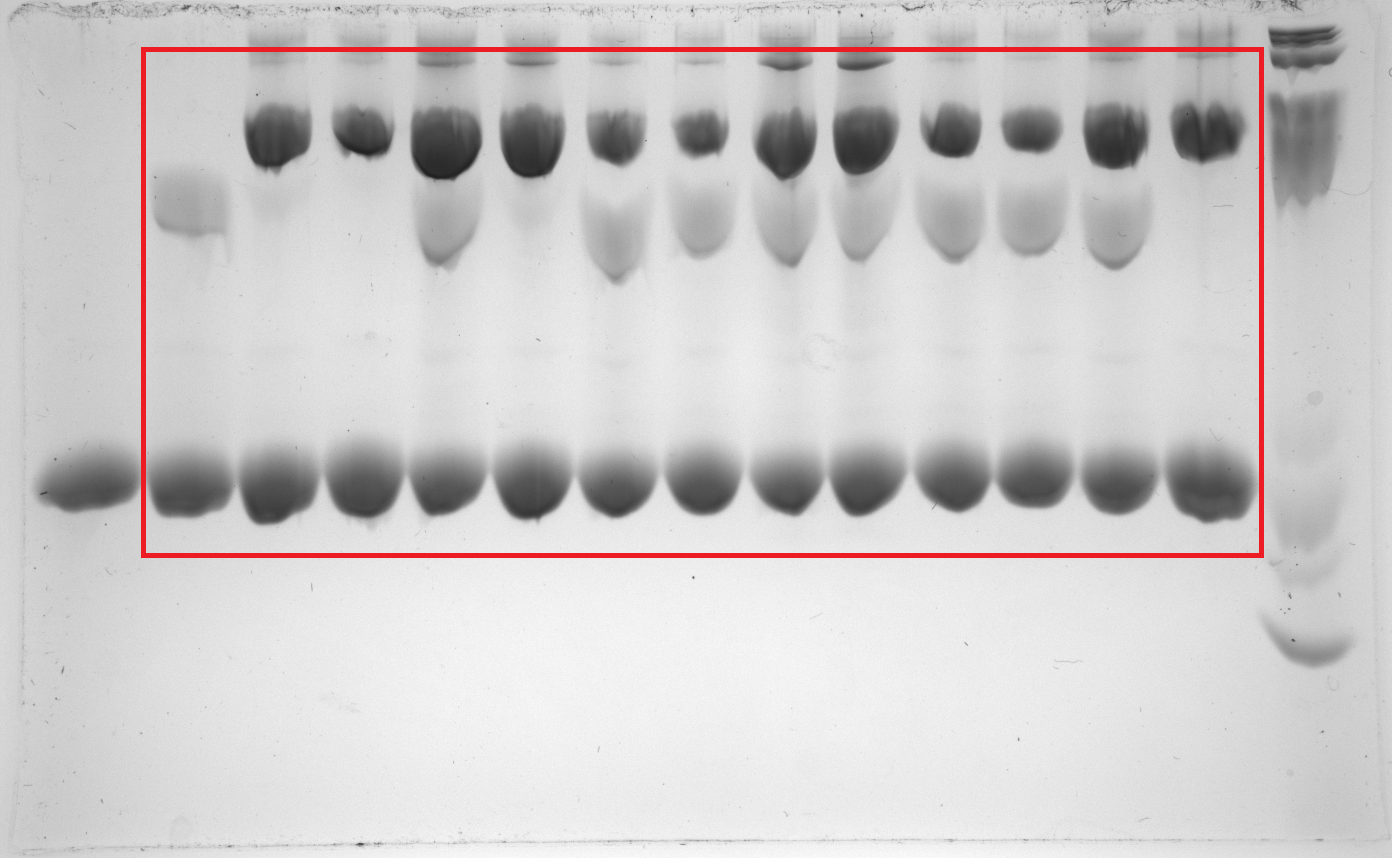

Supplement: Supplementary file 6 — Source Data [file 41467_2021_24676_MOESM6_ESM.zip › Figure 3 gels/3E.tif]

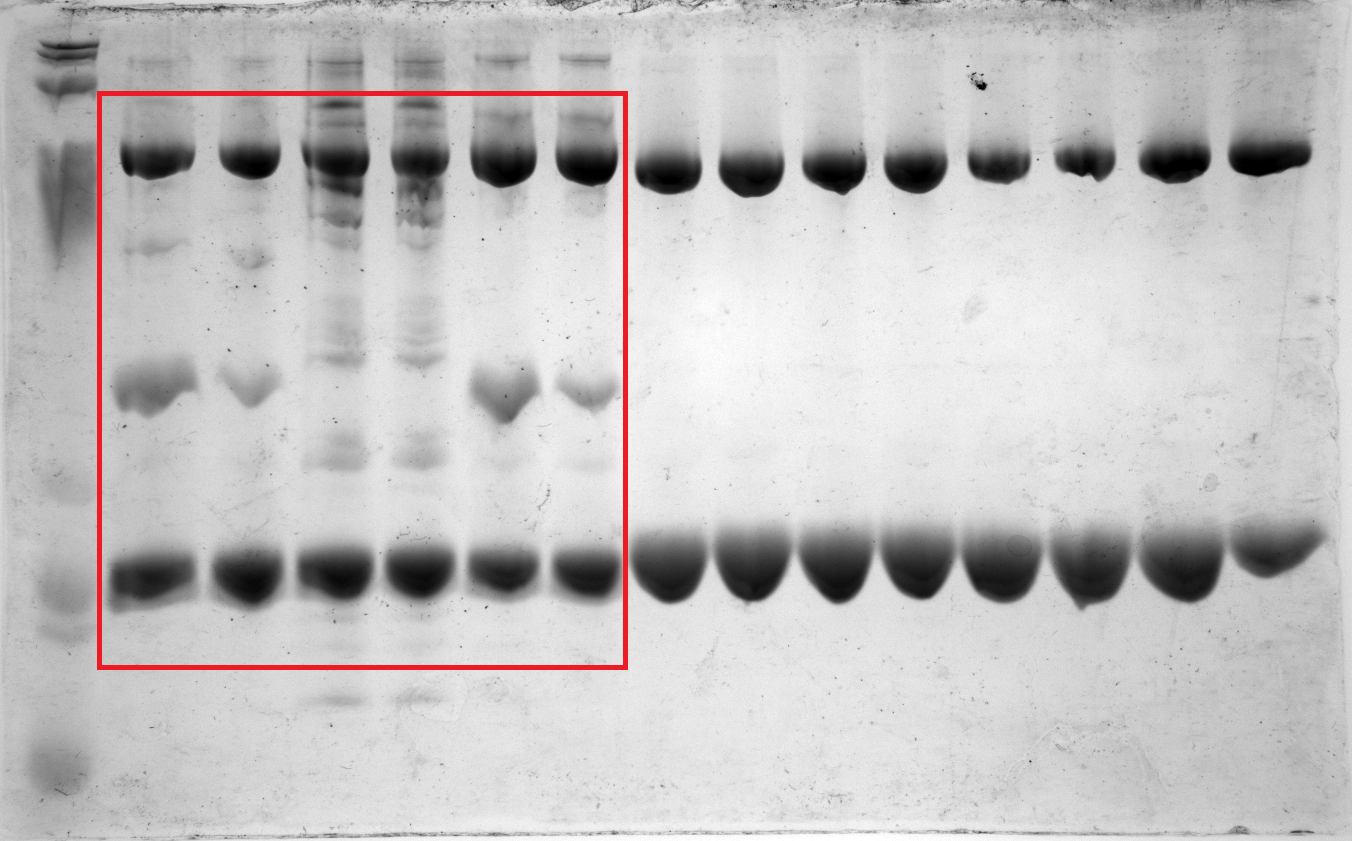

Supplement: Supplementary file 6 — Source Data [file 41467_2021_24676_MOESM6_ESM.zip › Figure 3 gels/3F.tif]

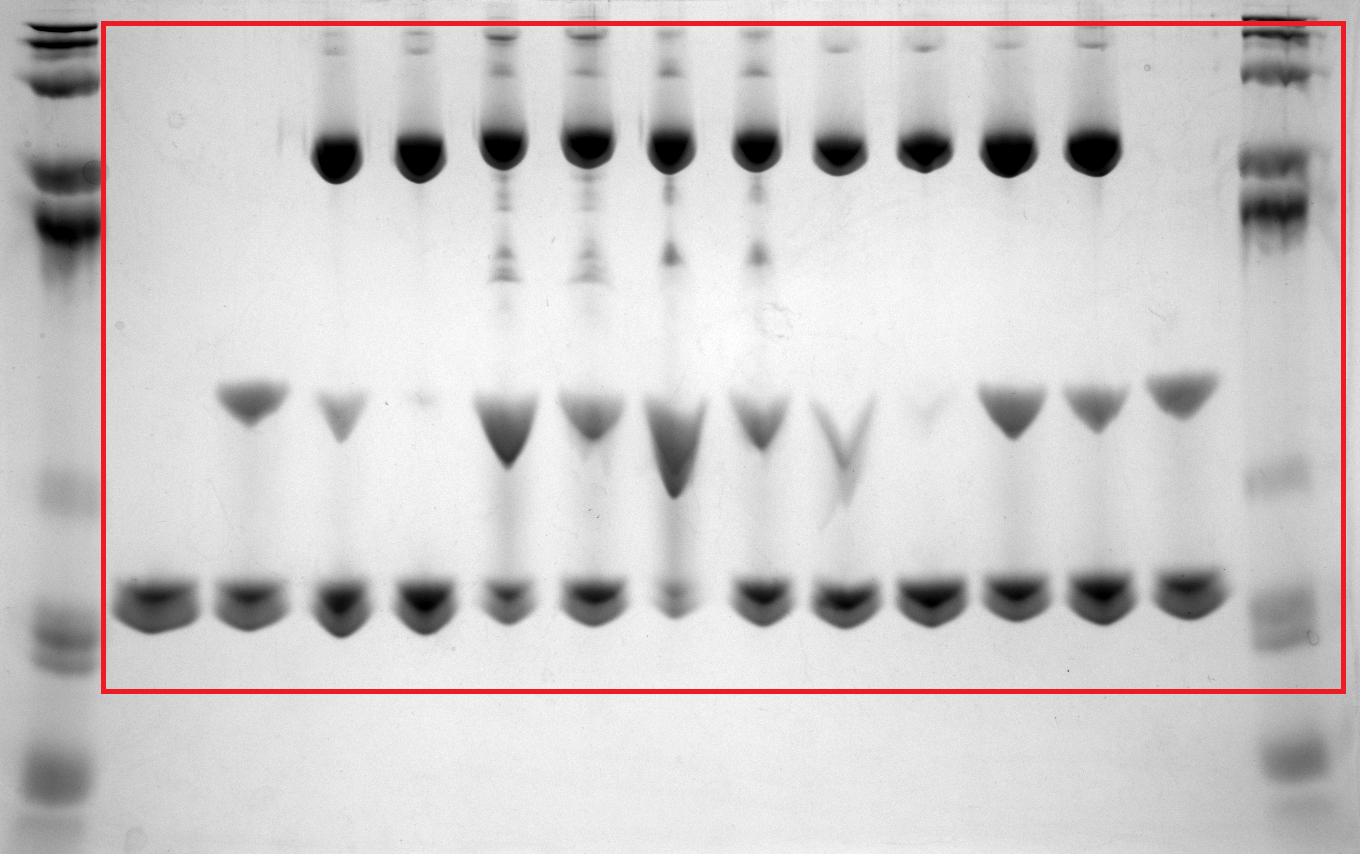

Supplement: Supplementary file 6 — Source Data [file 41467_2021_24676_MOESM6_ESM.zip › Supplementary Figure 4 gels/S4A.tif]

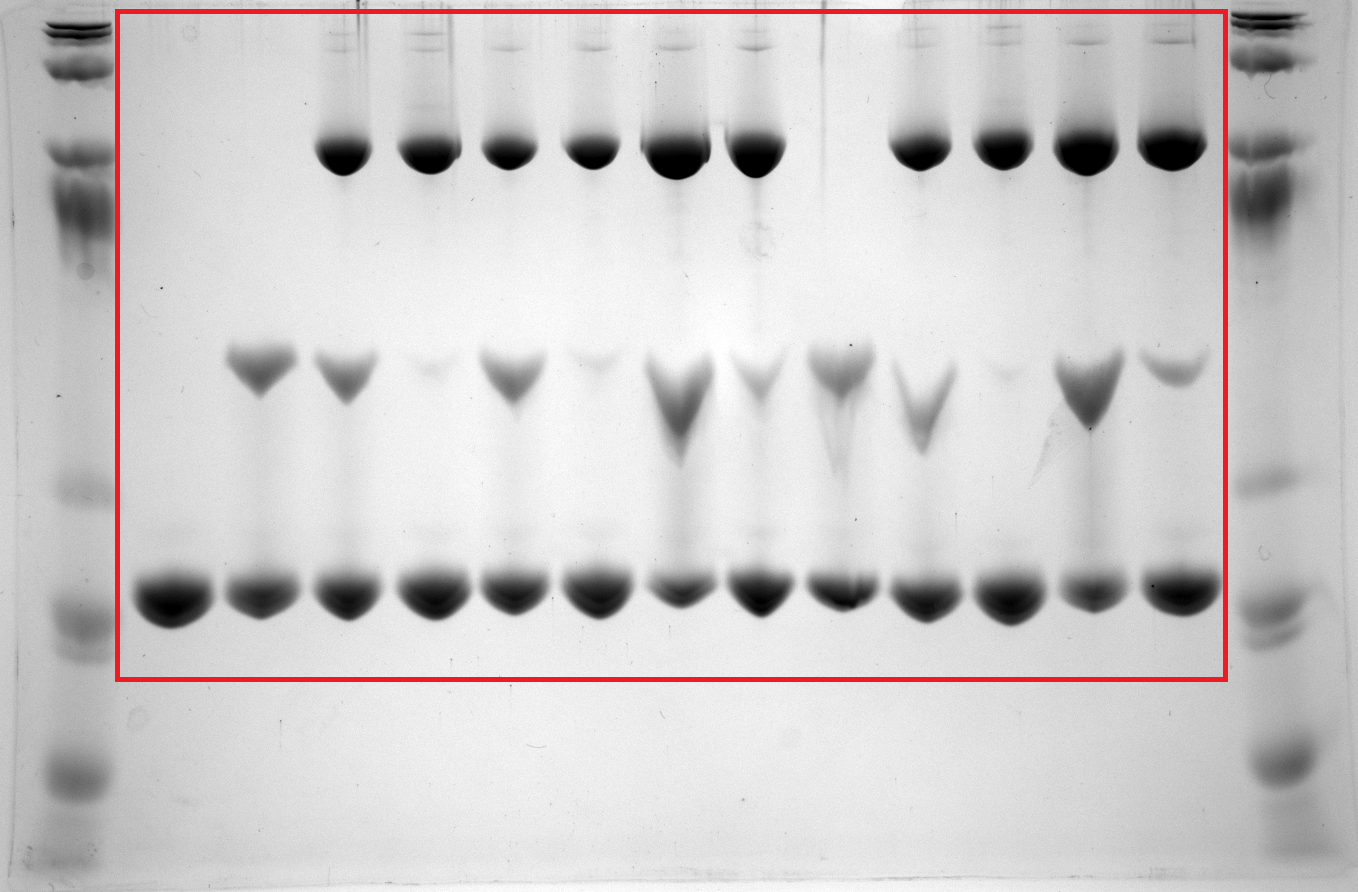

Supplement: Supplementary file 6 — Source Data [file 41467_2021_24676_MOESM6_ESM.zip › Supplementary Figure 4 gels/S4B.tif]

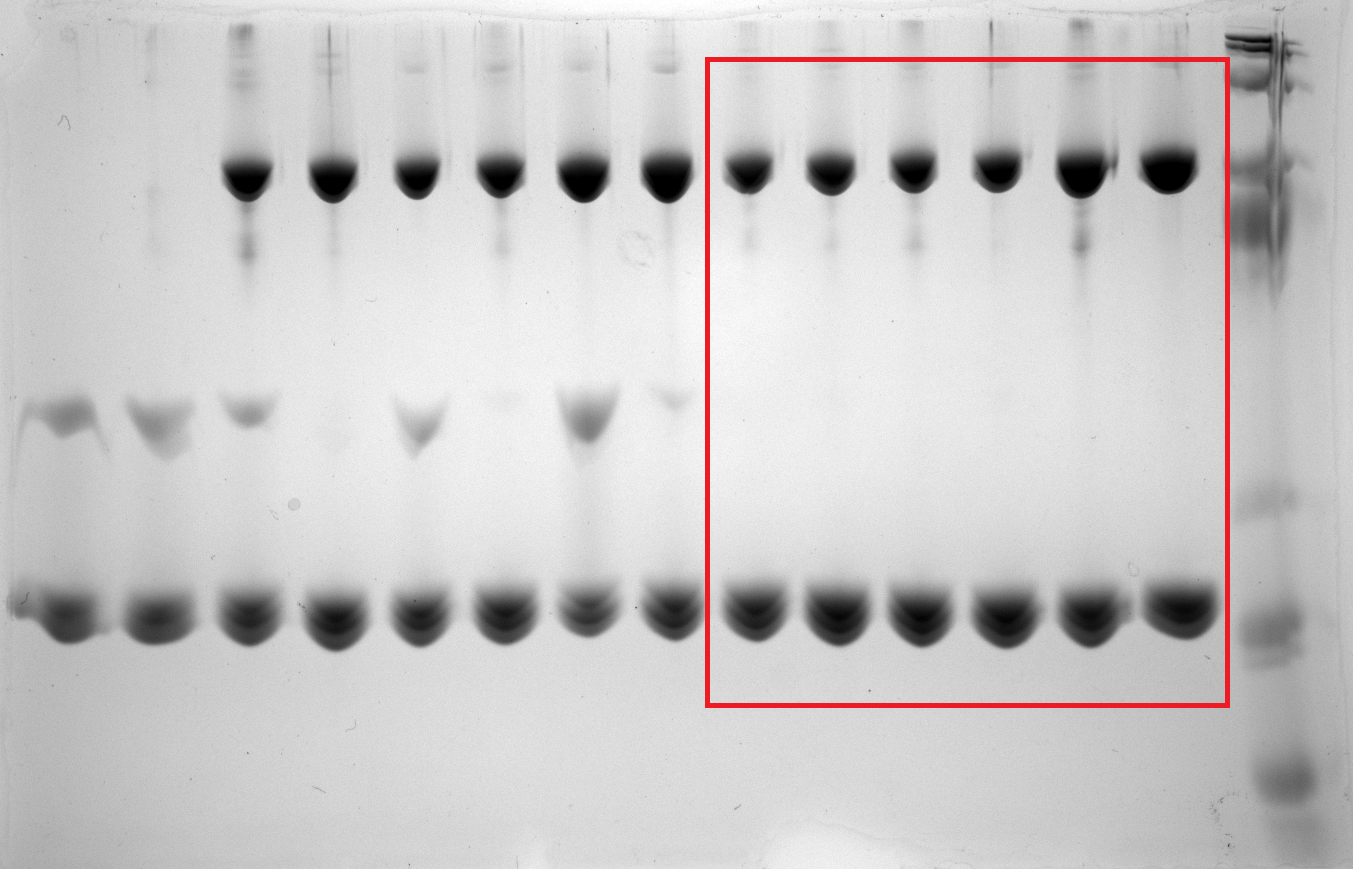

Supplement: Supplementary file 6 — Source Data [file 41467_2021_24676_MOESM6_ESM.zip › Supplementary Figure 4 gels/S4C.tif]

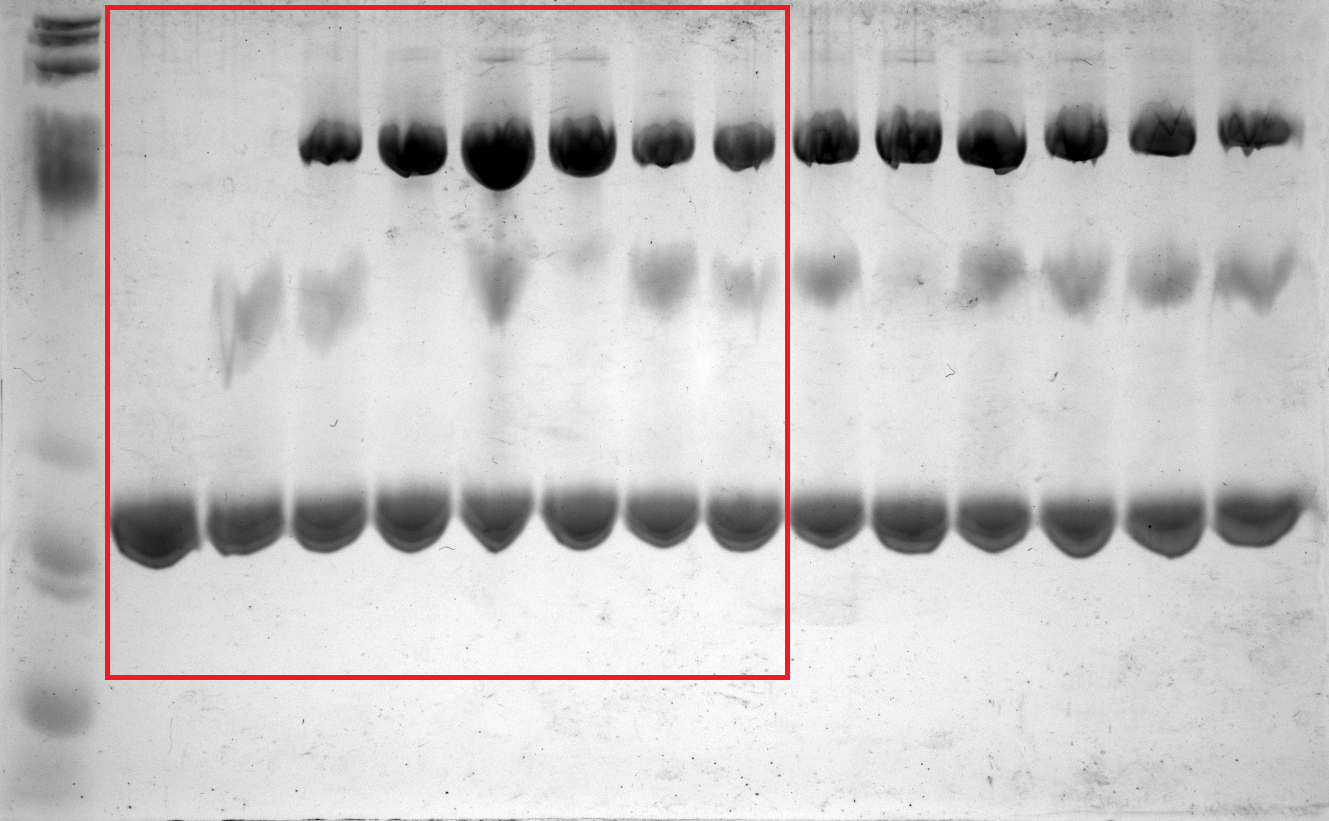

Supplement: Supplementary file 6 — Source Data [file 41467_2021_24676_MOESM6_ESM.zip › Supplementary Figure 4 gels/S4D.tif]

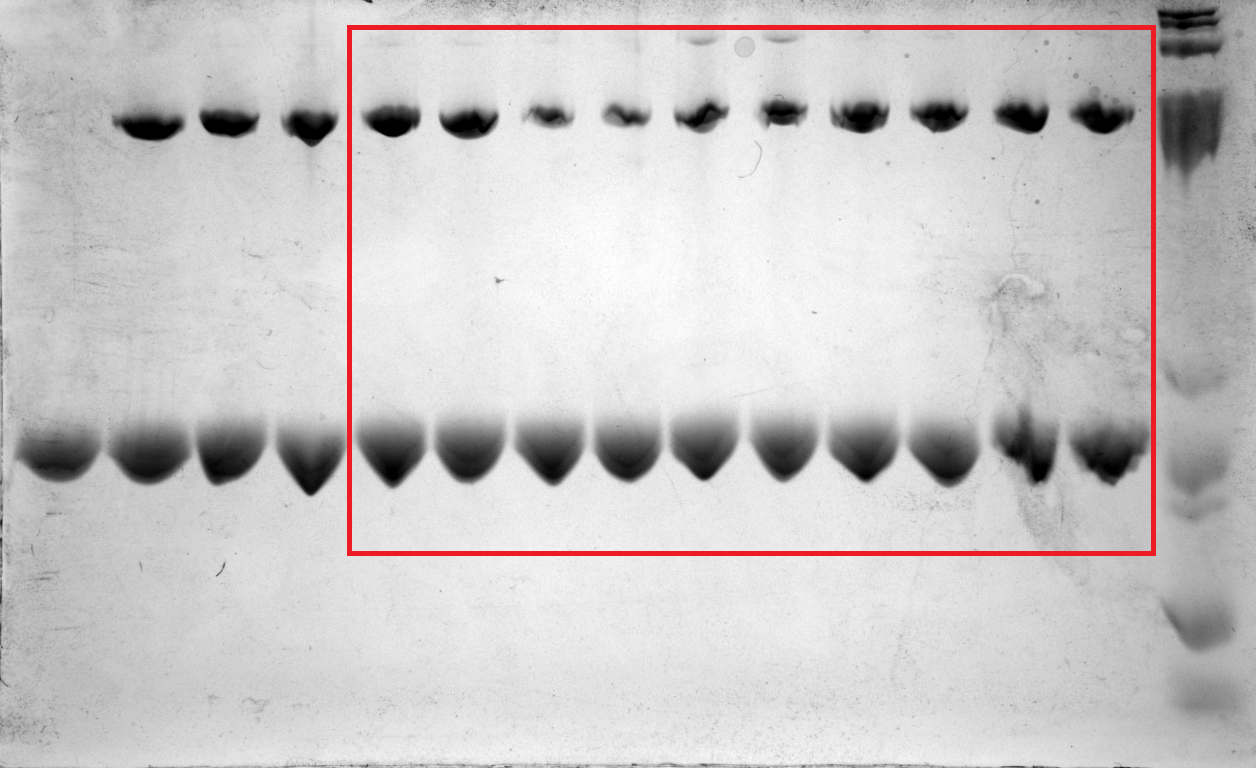

Supplement: Supplementary file 6 — Source Data [file 41467_2021_24676_MOESM6_ESM.zip › Supplementary Figure 4 gels/S4E.tif]

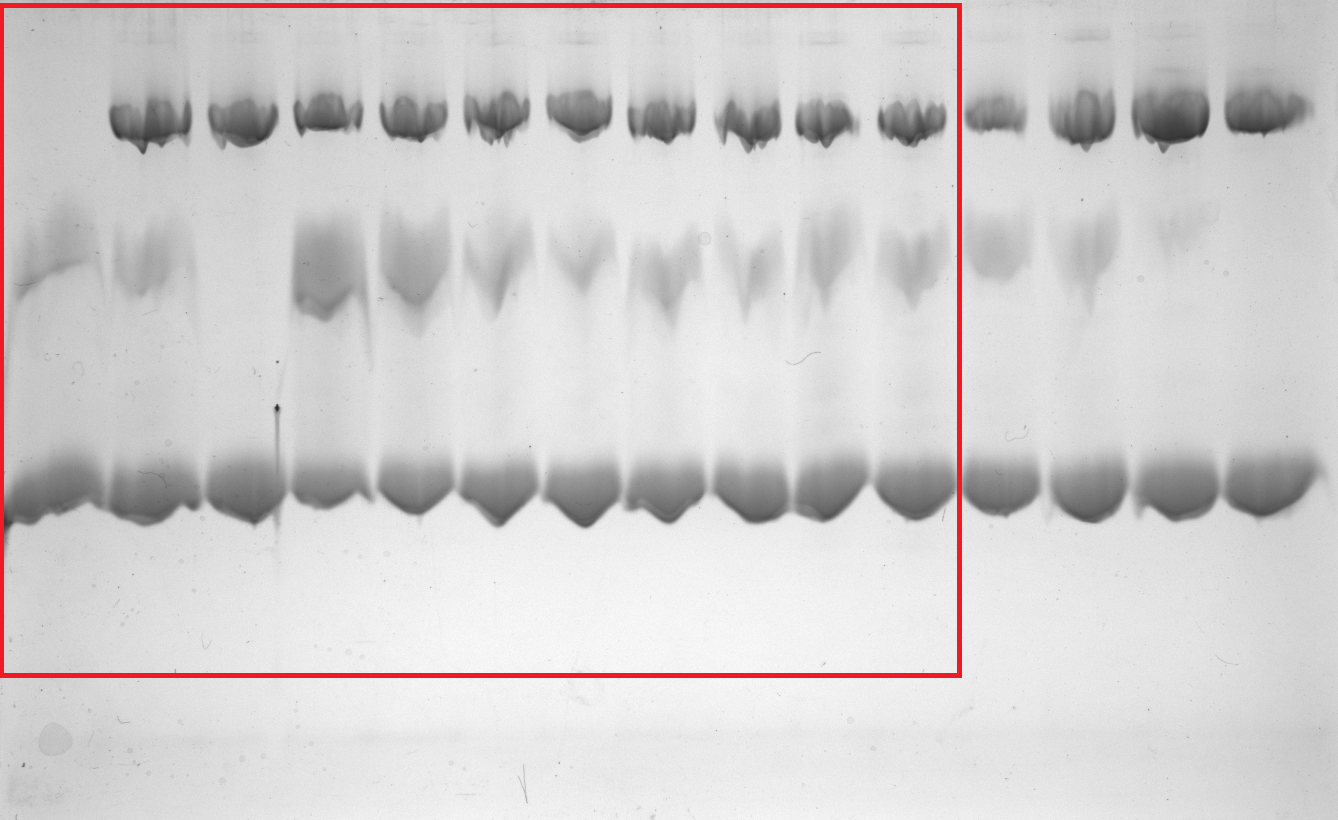

Supplement: Supplementary file 6 — Source Data [file 41467_2021_24676_MOESM6_ESM.zip › Supplementary Figure 4 gels/S4F.tif]

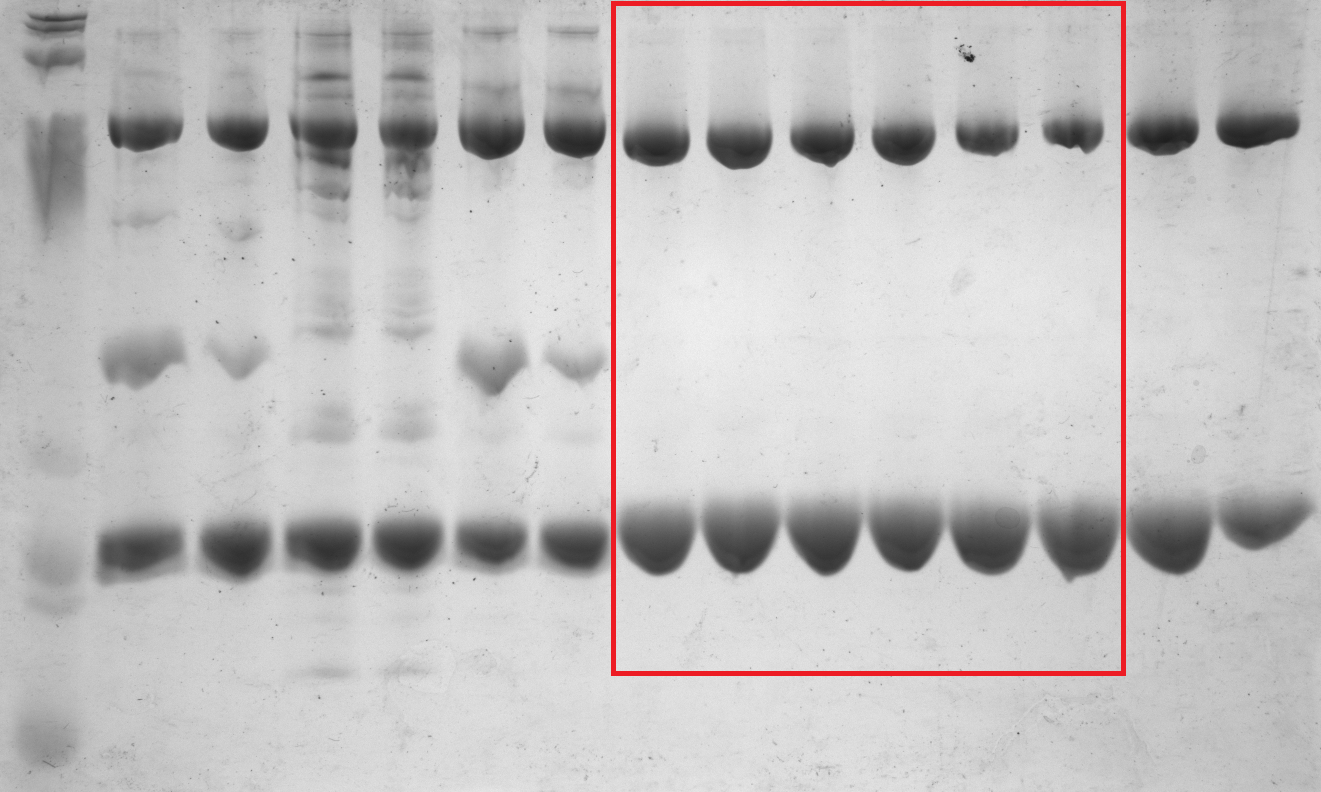

Supplement: Supplementary file 6 — Source Data [file 41467_2021_24676_MOESM6_ESM.zip › Supplementary Figure 4 gels/S4G.tif]

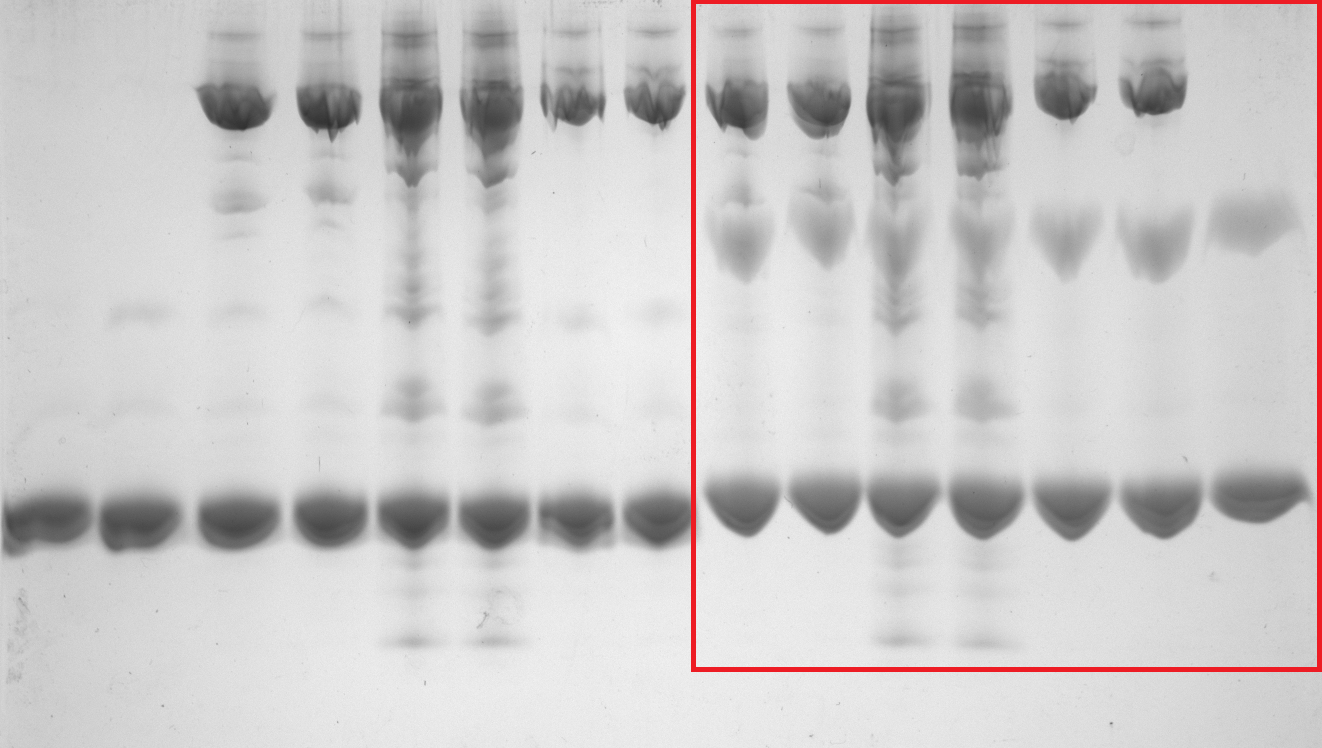

Supplement: Supplementary file 6 — Source Data [file 41467_2021_24676_MOESM6_ESM.zip › Supplementary Figure 4 gels/S4H.tif]

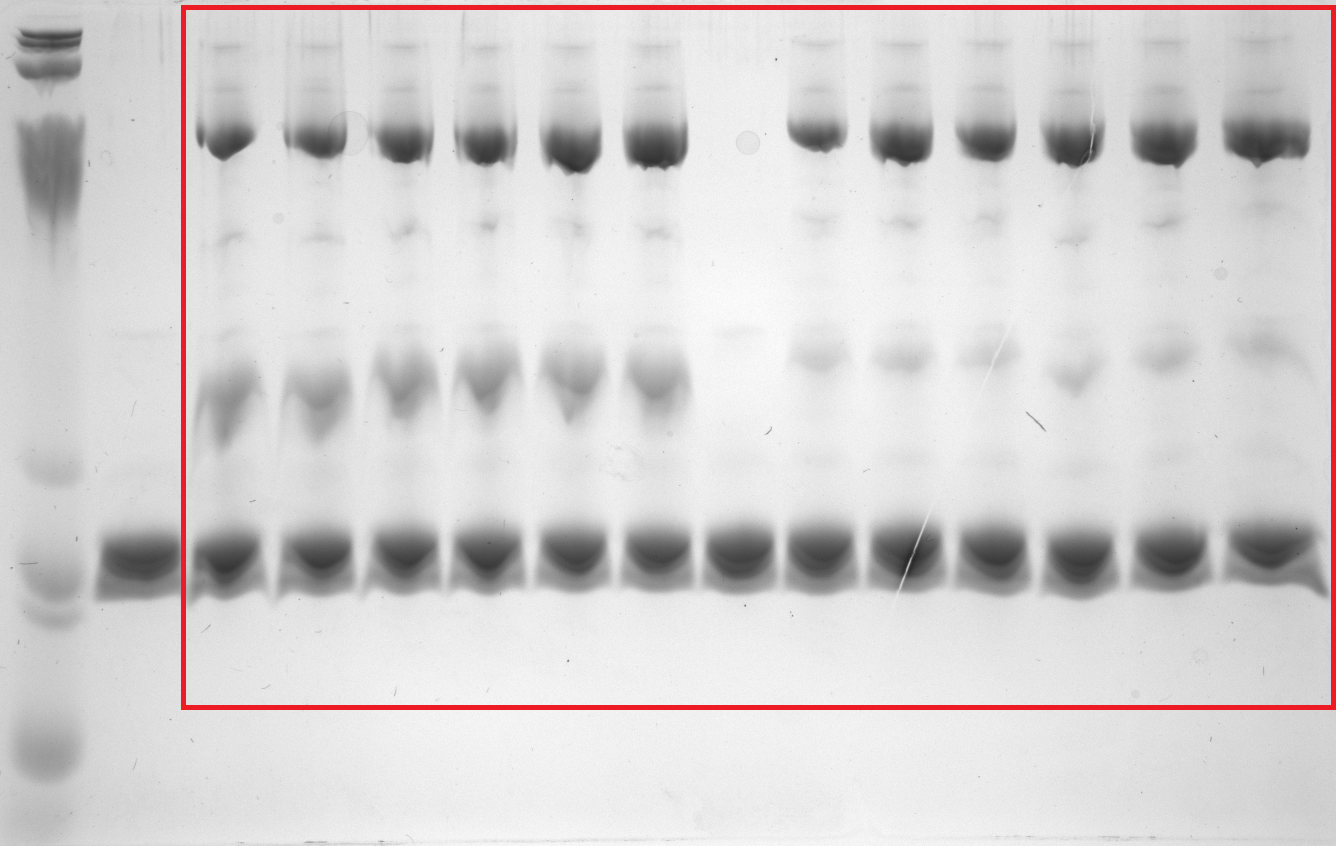

Supplement: Supplementary file 6 — Source Data [file 41467_2021_24676_MOESM6_ESM.zip › Supplementary Figure 4 gels/S4I.tif]
